# Altitude hypoxia and hypoxemia: pathogenesis and management

**DOI:** 10.1038/s41392-025-02531-1

**Published:** 2026-01-23

**Authors:** Chunmei Cai, Guohua Ni, Lei Chen, Cheng Deng, Shengjun Chai, Rui Wang, Rong Zhang, Fengming Luo, Ri-Li Ge

**Affiliations:** 1 Research Center for High Altitude Medicine, School of Medical, Qinghai University, Xining, Qinghai PR China; 2 Center for High Altitude Medicine, West China Hospital, Sichuan University, Chengdu, Sichuan PR China

**Keywords:** Cardiovascular diseases, Respiratory tract diseases

## Abstract

At high altitudes, which typically exceed 2500 m, approximately 80 million people reside permanently, with over a million visitors annually. The primary effect of high altitude is hypobaric hypoxia, which leads to decreased oxygen availability and a cascade of physiological responses. However, inadequate or excessive responses can lead to malacclimatization, resulting in hypoxemia and various high-altitude illnesses, including acute mountain sickness (AMS), high-altitude cerebral edema (HACE), high-altitude pulmonary edema (HAPE), chronic mountain sickness (CMS), and high-altitude pulmonary hypertension (HAPH). Acute altitude illnesses (AMS, HACE, and HAPE) stem from inadequate acclimatization, whereas chronic conditions (CMS and HAPH) reflect prolonged or excessive adaptive responses. This review briefly summarizes the current knowledge on the clinical manifestations, epidemiology, and risk factors for high-altitude diseases. Additionally, this review systematically discusses the most recent pathophysiological mechanisms underlying these conditions, with a special emphasis on genetic susceptibility and chronic altitude illness (CMS and HAPH). Furthermore, a comprehensive overview of current prevention and treatment strategies is provided, emphasizing the promising effects of natural medicines, especially traditional Tibetan medicines. Despite extensive research, the exact mechanisms underlying these illnesses remain elusive, and options for their management are still limited. This review aims to provide novel insights into the pathogenic mechanisms of these complex conditions and guide future research directions to improve the prevention and management of high-altitude illnesses.

## Introduction

At high altitudes, which typically exceed 2500 m, approximately 80 million people reside permanently, with over one million visitors annually for tourism, sports, religion or work.^1^ The largest populations of permanent residents at high altitudes are found in the Andes of South America, the Qinghai-Tibet Plateau, the Caucasus of Eastern Europe, Ethiopia, and the Himalayas. In recent years, an increasing number of people, especially the older population, have been drawn to such areas, including trekkers, climbers, miners, military personnel, astronomers, and athletes undergoing sports training.^2^ Both native highlanders and newcomers are at risk of high-altitude disease. The incidence and severity of high-altitude disease are determined by the attained altitude, ascent variables (including environmental and behavioral factors), and individual susceptibility.^3^ Owing to these factors, as well as diverse study designs and biases, the exact prevalence of altitude sickness among unacclimatized lowlanders and highlanders remains uncertain.

The major effect of high altitude on human physiology is a decrease in partial oxygen pressure and content in the circulating blood, which is directly associated with the reduction in barometric pressure that occurs with ascent.^4^ Hypoxia, i.e., diminished oxygen availability, is defined as a decrease in the partial pressure of oxygen compared with normal status in the blood, organs, tissue, or cells.^5^ Hypoxic stress at high altitude can trigger a series of physiological responses across multiple organ systems, especially the brain (increased cerebral blood flow), pulmonary system (increased ventilation and pulmonary vascular remodeling), cardiovascular system (increased heart rate, cardiac output, and systemic blood pressure), renal system (increased bicarbonate excretion and erythropoietin (EPO) secretion), and hematologic system (increased red cell mass, hemoglobin (Hb) concentration, and hematocrit (Hct)), thereby increasing oxygen content and delivery in the body.^6,7^ These compensatory adaptive changes are collectively referred to as acclimatization, which enables individuals to work and live at high altitude without any discomfort. An inadequate or excessive compensatory adaptive response, termed malacclimatization, can impair oxygen delivery, leading to hypoxemia, which is defined as a decrease in the arterial partial pressure of oxygen (PaO₂).^8^ Hypoxemia is a well-known primary cause of high-altitude illness among many visitors, sojourners, and natives.^8,9^ High-altitude hypoxia-related diseases include acute mountain sickness (AMS, also known asacute mild altitude disease (AMAD)), high-altitude cerebral edema (HACE), high-altitude pulmonary edema (HAPE), chronic mountain sickness (CMS), high-altitude pulmonary hypertension (HAPH; also known as high-altitude heart disease, HAHD), high-altitude deterioration (HAD), etc.^6,10^ Acute altitude illnesses (e.g., AMS, HACE, and HAPE) result from inadequate physiological adaptation to hypobaric hypoxia, whereas chronic conditions such as HAPH and CMS reflect the pathological consequences of prolonged or excessive adaptive responses.

**Fig. 1:**
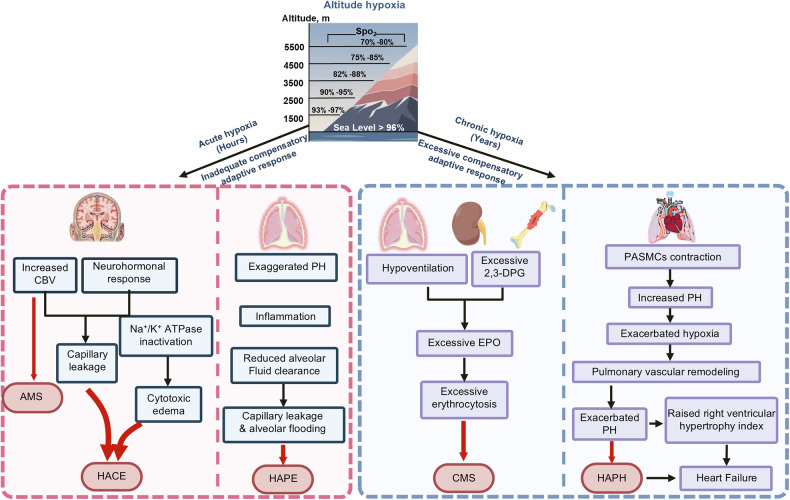
Classification of high-altitude diseases. Acute altitude illnesses (onset within h, representing a failure of physiological adaptation) encompass AMS, HACE, and HAPE. An increased CBV, enhanced neurohormonal activation, and decreased Na⁺/K⁺ ATPase activation contribute to capillary leakage and cytotoxic edema in HACE pathogenesis. HAPE is driven by exaggerated pulmonary hypertension, inflammation, and impaired alveolar fluid clearance, causing capillary leakage and alveolar flooding. Chronic altitude disorders (onset over years, resulting from pathological overadaptation) include CMS and HAPH. CMS arises from hypoventilation and excessive 2,3-DPG, resulting in excessive erythropoietin (EPO) production and subsequent excessive erythrocytosis. HAPH progresses via PASMCs contraction-mediated HPV, which further exacerbates hypoxia to trigger HPVR, ultimately leading to exacerbated pulmonary hypertension, right ventricular hypertrophy, and heart failure. PH, pulmonary hypertension

Within h to 5 days of exposure to hypobaric hypoxia, some individuals are susceptible to acute altitude illness, including mild (AMS) and severe (HAPE and HACE) forms (Fig. 1).^11^ The symptoms of AMS, a self-limiting and nonfatal disease, are usually nonspecific and include headache with any of the following: poor appetite or nausea/vomiting, fatigue or lassitude, and dizziness/light-headedness.^12^ AMS can progress to a potentially lethal illness, HACE, associated with neurological signs, such as truncal ataxia, altered mental status, depressed consciousness, and encephalopathy.^13^ HAPE, a noncardiogenic pulmonary edema and the most fatal form of acute altitude illness, is characterized by dyspnea, loss of stamina, and dry cough, followed by dyspnea at rest, cyanosis, gurgling in the chest and pink frothy sputum.^14^ Natives or long-term high-altitude sojourners, who are chronically exposed to altitude hypoxia, may develop several altitude-related illnesses, including polycythemia, systemic hypertension, pulmonary hypertension, congenital heart disease, thromboembolic disease, lung disease, mental deterioration, as well as pregnancy and neonatal problems.^15^ There is a consensus that CMS (or Monge’s disease) and HAPH are the most common forms of chronic altitude illness (Fig. 1). CMS, which is more prevalent among Andeans than Tibetans, is characterized by excessive erythrocytosis (females Hb ≥19 g/dL, males Hb ≥21 g/dL) and severe hypoxemia, and is frequently associated with neurological symptoms, pulmonary hypertension, and impaired pulmonary ventilation/perfusion.^15,16^ HAPH, which occurs in high-altitude adults, is defined by a mean pulmonary artery pressure (mPAP) > 30 mmHg or a systolic pulmonary artery pressure (sPAP) > 50 mmHg, and is generally associated with right ventricular hypertrophy, heart failure, moderate hypoxemia, and the absence of excessive erythrocytosis.^15^ HAD symptoms broadly include weight loss, poor appetite, slow recovery from fatigue, lethargy, irritability, lack of willpower to start new tasks, slowed mental processes, dull affect, and impaired cognitive function.^17^ Currently, there is still a lack of consensus regarding the diagnosis or management of HAD, making it challenging to accurately delineate its prevalence and pathogenic mechanisms.

To conquer the mountains and exploit resources, research on high altitude-related sickness rapidly developed. John B West has skilfully documented the history of high altitude medicine and physiology, highlighting key observations, experiments, and challenges.^18^ Although AMS has been known for more than a century, its diagnostic standard was only established at the 1991 International Hypoxia Symposium in Banff, Canada. Recent studies have revealed that “difficulty sleeping”, one of the five symptoms scored for AMS, is more of an effect of hypoxia than closely related to other AMS symptoms, thereby removing the sleep component from the Lake Louise AMS score in 2018.^12^ HACE was originally described by Ravenhill et al. and officially named by Fitch.^19^ The 2024 Wilderness Medical Society (WMS) Practice Guidelines updated the definition of HACE as severe AMS with neurological signs.^20^ HAPE, often misdiagnosed as acute pneumonia before 1960, was first reported by Dr Charles Houston^21^ and later identified as noncardiogenic pulmonary edema by Hultgren et al. ^22^ At the 1998 meeting of the International Society of Mountain Medicine (ISMM) in Matsumoto, Japan, an international consensus statement on CMS was developed. CMS was first described by Carlos Monge in 1928. A more recent consensus statement was developed at the 2004 ISMM meeting in Xining, China, in which the Qinghai CMS score enables quantitative assessment of CMS severity and comparison of CMS cases within or among different countries.^15^ HAPH was first documented by Wu and Liu in 1955, and since then, Chinese investigators have extensively used this term. It is necessary to differentiate HAPH from subacute mountain sickness (SAMS), which is rarely seen in adults but mainly occurs in infants.^23^

With an increasing global demand for recreation and habitation at high altitudes, people, especially travelers, altitude residents, physicians, or paramedical personnel, need to be familiar with symptom recognition and the pathogenic mechanisms of high altitude-related sickness, so that appropriate and novel prevention and treatment methods can be adopted to reduce the severity or morbidity. In this narrative review, we briefly summarize the clinical characteristics, major events, and epidemiology of high-altitude disease; focus on the pathophysiological changes, genetic susceptibility and potential mechanisms involved in altitude hypoxia-related sickness; and detail the preventive and therapeutic advances in animal experiments, clinical trials, and clinical experience.

## Epidemiology and risk factors for altitude illness

### Acute altitude illness

The primary determinant of high-altitude hypoxia-related sickness is the altitude reached.^24^ Since HACE is rarely reported, systematic analysis of its risk factors is still lacking.^25^ HACE often begins as a severe form of AMS, suggesting that the determinants of HACE may be similar to those of AMS.^20,26^ The major risk factors for AMS, HACE, and HAPE include altitude attained (especially the sleeping altitude), ascent rate, individual susceptibility, and degree of preacclimatisation.^25,27^ Other risk factors for high-altitude illness include preexisting diseases, cold weather, fatigue, exercise, obesity, sex, and age.^9^ Several preexisting diseases, particularly cardiopulmonary diseases (such as pulmonary hypertension and bleeding disorders), may increase susceptibility to high-altitude disease.^28^ Globally, the reported prevalence of AMS, HACE, and HAPE varies significantly due to differences in these confounding variables, as well as study designs and biases.

The prevalence of AMS generally depends on the altitude attained and speed of ascent. The incidence of AMS increases significantly with elevation. At 2000 m, only 12% of the subjects exhibited symptoms^29^; at 3050 and 3506 m, 75–79% of the unacclimatized persons experienced symptoms^30^; and at 3800 and 4310 m, nearly all the individuals experienced some symptoms.^31^ In the Western Alps, the AMS incidence was 9%, 13%, 34%, and 53% at altitudes of 2850, 3050, 3650, and 4559 m, respectively, whereas in the Eastern Alps, it was 6.9%, 9.1%, 17.4%, and 38.0% at 2200, 2500, 2800, and 3500 m, respectively.^32,33^ In addition, the AMS prevalence is positively correlated with the rate of ascent. When individuals ascended to similar altitudes, gradual climbing allowed for partial acclimatization, resulting in a lower incidence (50% versus 84%) and less severity.^30^ The differences in susceptibility to AMS between males and females remain unclear, with conflicting reports. Hou et al. revealed that females tend to develop AMS,^34^ whereas others reported that men were slightly more affected by AMS than women.^35^ AMS is more common in children and younger individuals than older people aged >40–60 years,^36,37^ although a recent meta-analysis revealed no association between age and AMS risk.^38^ The impact of exercise on the occurrence and severity of AMS is also contradictory.^39–41^ Interestingly, some evidence has shown that smoking slightly decreases the risk of AMS and protects against AMS development.^42,43^ In addition, the prevalence of AMS is obviously higher in subjects with patent foramen ovale (PFO), an embryologic residual right-to-left cardiac shunt that can impair pulmonary gas exchange efficiency, than without PFO.^44,45^ Even after acclimatization to altitude hypoxia, individuals with PFO exhibited poorer gas exchange efficiency and a more blunted ventilatory response.

HACE is much rarer than AMS and rarely occurs below 4000 m. At ~4000 m, HACE affects approximately 0.28–1% of people, including trekkers, sojourners, workers, climbers, and soldiers.^27,30,46^ Given that most individuals at very high altitudes are males, determining sex differences in both HACE incidence and HAPE incidence is difficult. HAPE rarely occurs below 3000 m and appears to occur frequently in children and younger adults.^47,48^ There are two populations affected by HAPE: unacclimated lowlanders and acclimatized residents returning from low altitudes (re-entry). A prospective cohort study revealed a HAPE incidence of 1.7% among 1326 people ascending to about 4000 m.^27^ Remarkably, rapid ascent to 4500 or 5500 m significantly increases HAPE incidence compared with slow ascent (0.2% versus 7% or 2.5% versus 15.5%, respectively), with a recurrence risk of ~60% under rapid ascent conditions.^14,49^

In summary, the contributions of several risk factors to acute altitude sickness yield controversial results, and it is still unclear whether and how some determinants affect the occurrence and development of acute mountain illness, especially HACE and HAPE; thus, further comprehensive investigations are needed.

### Chronic altitude sickness

#### CMS

The prevalence of CMS varies by altitude, lung disease, sex, age, sleep apnea, obesity, and genetic factors. The reduction in atmospheric oxygen content due to increasing altitude is a fundamental factor in CMS pathogenesis. Studies have shown an increase in the incidence of CMS with increasing altitude.^50,51^ On the Qinghai-Tibet Plateau, the CMS prevalence rates are 1.05%, 3.75%, and 11.83% at altitudes of 2261–2980, 3128–3980, and 4000–5226 m, respectively.^50^ Similarly, in Himachal Pradesh, India, no CMS cases were observed at 2350–3000 m, whereas the number of cases rose to 13.3% at 3000–4150 m.^51^ Insufficient ventilation caused by various factors, such as preexisting lung diseases, sleep-disordered breathing, and smoking, can also affect the incidence of CMS. The harsh high-altitude environment, characterized by low ambient oxygen and low temperatures, can exacerbate preexisting lung diseases such as chronic bronchitis, emphysema, asthma, and obstructive lung disease, leading to hypoxemia and secondary polycythemia.^52^ A study in Cerro de Pasco (4300 m) showed higher CMS scores and Hb concentrations but lower SaO_2_ and peak expiratory flow rate (PEFR) values in a chronic lower respiratory disease (CLRD) group than normal.^53^ Importantly, the CMS incidence was substantially higher in individuals with respiratory diseases, especially CLRD (32.4%), than in normal individuals (11.3%). In Bolivia, the CMS incidence was reported to be between 6% and 8% among the male population of La Paz (3600 m),^54^ whereas a hospital study in the same region reported a frequency of 28%, predominantly among patients with respiratory diseases.^55^

Sleep-disordered breathing also appears to aggravate CMS by affecting respiratory function. In the Yushu state of the Qinghai-Tibet Plateau (3780 m), CMS patients had significantly lower SaO_2_ levels than controls, with CMS scores positively related to the apnea-hypopnea index and negatively correlated with the SaO_2_ value.^56^ Concurrently, Peruvian investigators also reported a lower mean sleep-time pulse O_2_ saturation (SpO_2_) and greater percentage of sleep time with SpO_2_ < 80% in CMS patients in Peru (4340 m) than in healthy highlanders.^57^ The presence of PFO in CMS patients may further aggravate sleep-disordered breathing, contributing to more severe hypoxemia.^58^ Smoking is another risk factor for CMS at high altitudes. Ge et al. found that heavy smokers (20 cigarettes per day for 15 years) had lower mean forced expiratory flow during the middle half of the forced vital capacity (FEF25–75%) and SaO_2_ levels, while higher Hb levels than non-smokers, suggesting that smoking may induce excessive polycythemia due to increased carboxyhemoglobin and hypoxemia.^59^ The prevalence of polycythemia among smokers was approximately 3 times higher compared to non-smokers.^59^ Interestingly, CMS patients with cobalt toxicity exhibited greater polycythemia, indicating the contribution of cobalt to CMS progression.^60^

Gender differences were also observed, with CMS being more prevalent in men. In Lhasa (3,568 m), no cases of CMS were observed in 160 females, whereas 42 cases were observed in 579 males (7.3%).^61^ Similarly, all 27 reported cases of CMS from the western Himalayas were in males.^62^ The prevalence of CMS increases with age. In the Andean population of Cerro de Pasco (4340 m), the prevalence was 15.4% among men aged 30–39 years, increasing to 33% by the age of 60.^63^ Another study at the same location reported that CMS prevalence rose from 6.8% in the youngest age group (20-29 years) to 33.7% in the oldest age group (60–69 years).^64^ Notably, significant variation across different high-altitude populations is primarily attributed to ethnic differences, considering similar altitudes and uniform diagnostic criteria. Tibetans and Ethiopians, with a longer history of high-altitude residence, are considered to be more adapted in comparison to Andeans and Han immigrants, reflected by lower Hb concentrations and thus lower prevalence rates of CMS.^65^ Epidemiological studies on the Qinghai-Tibet Plateau revealed a CMS prevalence of 5.6% among Chinese Han immigrants, markedly higher than the 1.2% reported in the Tibetan native population.^50,66^ In addition, in La Paz (3883 m), Bolivia, 42 men (7%) were diagnosed with CMS.^67^ Most of the patients were elderly and obese, indicating that the obesity could be a significant risk factor for CMS. Further research by Ge et al. reported a positive correlation between body mass index (BMI) and CMS score.^68^

#### HAPH

Owing to variations in populations, diagnostic methods, and diagnostic criteria, the actual prevalence of HAPH among long-term high-altitude individuals is challenging to determine. Wu et al. reported a HAPH prevalence of 0.31% among 20,315 native adult Tibetans dwelling in Qinghai Province (2261–5188 m), China, using electrocardiogram (ECG), indicating its rarity in high-altitude residents.^69^ Later, studies in South America reported that the HAPH prevalence ranged from 5% to 18% in populations at Altiplano ( ≥ 3,200 m).^70^ In the Kyrgyzstan Himalayan population (2800–3100 m), Aldashev and colleagues reported an 18% prevalence of HAPH among 741 high-altitude residents based on ECG evidence.^71^ However, Negi et al. found a 3.23% prevalence via ECG among 1,087 subjects from Spiti Valley in India (3000–4200 m), another ethnic population in the Himalayas.^72^ These discrepancies could be attributed to the insufficient sensitivity (20%–59%) of ECG.^73^ Alternatively, the echocardiography has improved sensitivity and specificity (70% and 88%, respectively) for estimating mPAP via pulmonary artery acceleration time (PAAT), which measures the time from the onset of right ventricular ejection to the peak velocity across the pulmonary valve.^73^ Recently, Gou et al. carried out a cross-sectional study among 1129 native Tibetans in Ganzi Tibetan Autonomous Prefecture, China (3200 m), revealing a HAPH prevalence of 6.2%.^74^

Several factors, such as altitude, gender, age, ethnicity, and smoking, significantly influence HAPH incidence. HAPH incidence progressively increases with altitude. Wu et al. reported HAPH incidence of 0.04%, 0.48%, and 0.92% at 2261–2808, 3050–3797, and 4068–5188 m, respectively, among native adult Tibetans residing in Qinghai Province.^69^ Conversely, Negi et al. reported no significant correlation between the altitude of residence and HAPH prevalence in India’s Spiti Valley.^72^ Importantly, the HAPH prevalence may be inaccurate, possibly due to flawed diagnostic criteria adopted by Negi and colleagues, particularly the omission of excluding participants with erythrocythemia. Thus, the correlations identified by Negi et al. may lack reliability. Additionally, HAPH is more common in males than females according to studies conducted in South America.^70^ Similarly, Aldashev et al. reported a HAPH prevalence of 23% among 347 males and 6% among 394 females.^71^ Gou and colleagues also found a higher prevalence in males (8.6% among 440 males) than females (4.6% among 689 females).^74^ Notably, HAPH is more prevalent in elderly than young individuals. Gou et al. delineated rates of 2.85%, 6.01%, and 13.47% among subjects aged <40, 40–60, and >60 years, respectively.^74^ Compared to Chinese Han immigrants (1.55%), Tibetan natives had a lower prevalence (0.11%), indicating that Tibetans have better adaptation.^69^ Smoking may be another risk factor for HAPH at high altitudes. Aldashev et al. found a higher prevalence of smoking among males with HAPH (34%) than females (0%), suggesting the involvement of smoking in higher prevalence in males.^71^ Gou et al. revealed a higher percentage of smokers in HAPH group (5.1%) compared to control group (1.4%), though this difference was not statistically significant (p = 0.07).^74^ Hence, whether smoking is a risk factor for HAPH warrants further investigation. Gou and colleagues also found that HAPH was positively correlated with age, metabolic syndrome, male sex, obesity, and hypoxemia.^74^

## Pathophysiological changes, genetic susceptibility and pathogenic mechanisms in altitude illness

A rational approach to preventing and treating high-altitude illness requires comprehensive knowledge of pathophysiology. Altitude hypobaric hypoxia leading to hypoxemia triggers high-altitude sickness, although the precise mechanisms remain poorly understood. Although the pathophysiology of high-altitude illness is still in its infancy owing to its rarity, accumulating studies over the past decades have gradually revealed the involvement of multiple factors.

### Pathophysiological changes

#### Acute altitude illness

Since AMS is a self-limiting and nonlethal disease, its pathophysiology is relatively unexplored. HACE autopsy findings include brain weights of 1260–1730 g, cerebral vascular congestion, flattened gyri, narrowed sulci, multiple petechial hemorrhages, subarachnoid fluid accumulation, and hippocampal herniation or cerebellar tonsil herniation.^75^ Histology reveals interstitial edema and swollen neurons. CT scan shows diffuse low-density in subcortical areas, suggesting cerebral edema.^76^ Comparatively, another imaging technology, magnetic resonance imaging (MRI), is more valuable for identifying and differentially diagnosing HACE.^25^ Typical neuroimaging features of HACE on MRI include prominent hyperintense signals with diffuse microhemorrhages in the white matter and corpus callosum.^77^

In contrast, the pathology of HAPE is best understood among high-altitude illnesses for well-conducted autopsy studies. Necropsy studies show severe diffuse pulmonary edema with bloody foamy fluid in the cut surfaces, trachea, and bronchi.^78^ The total lung weight ranges from 1229 to 2370 g. Importantly, there is no evidence of lung infection or left ventricular failure, confirming that HAPE is neither a type of pneumonia nor cardiogenic pulmonary edema. Histological findings further show thrombosis in terminal pulmonary arterioles and capillaries, as well as hyaline membranes lining the walls of alveoli, indicating high protein exudate from extensive capillary injury.^79,80^ The alveolar spaces are filled with edema coagula containing varying amounts of fibrin, red blood cells (RBCs), monocytes, lymphocytes, neutrophils, macrophages, and polymorphs. Pulmonary artery pressure (PAP) markedly increases in this condition.^81^ The chest radiographs (X-ray) of HAPE patients are abnormal, with heterogeneous opacities in the lower and middle zones bilaterally, indicating pulmonary edema.^82^ CT scans of HAPE patients show numerous small, confluent airspace consolidations, indicating a patchy and peripheral distribution of edema.^83^

#### Chronic altitude illness

A hallmark of CMS is profound erythrocytosis, with markedly elevated Hb and Hct levels.^84^ This compensatory response to chronic hypoxia increases blood viscosity and impairs microcirculation. Although CMS is a systemic disease affecting multiple organ systems, direct mortality from this condition remains rare. Physical examination typically reveals cyanosis, particularly at the nail beds, ears, and lips, alongside clubbing fingers.^85^ Ocular manifestations include conjunctival hyperemia, capillary dilation, and watery eyes. Cardiac abnormalities in CMS are evident on ECG, showing right ventricular and right atrial hypertrophy, manifested as right axis deviation, peaked P waves in leads II/III/aVF and in right precordial leads, as well as T wave inversion.^50^ Chest X-ray reveals right-heart enlargement, with a prominent main pulmonary artery.^50^ Furthermore, functional MRI (fMRI) indicates increased gray matter volume but reduced white matter volume in CMS patients, alongside abnormal spontaneous activities in multiple brain regions.^86^ Polycythemia and hypoxemia further induce diffuse cerebral edema and sluggish cerebral blood flow on MRI images, leading to neurological complications.^87^ Autopsy findings in CMS patients show widespread pathological changes^68^: (1) Heart: enlarged volume/weight with dilated chambers filled with clots, myocyte necrosis, and endothelial swelling in myocardial capillaries; (2) Lungs: scattered hemorrhages, dilated pulmonary capillaries, and muscularization of arterial branches; (3) Brain: sulcal shallowing, vascular congestion, petechial hemorrhages, neuronal swelling, and interstitial edema; (4) Gastric mucosa: patchy hemorrhage and edematous changes.

Pulmonary hypertension is a universal compensatory response to altitude hypoxia, although it is often asymptomatic.^88^ Diagnosis relies on ECG, chest X-ray, echocardiography, pulmonary function test (PFT), pulmonary angiography (PA), and right heart catheterization.^89^ The ECG (e.g., right-axis deviation, P-pulmonale, RV hypertrophy) and chest X-ray (PA enlargement, right ventricular dilation) findings lack sensitivity in the early disease stage.^90^ Echocardiography, while superior for screening, may sometimes provide inaccurate assessments of PAP.^91^ It also serves a critical role in evaluating left heart function for discerning the etiology of pulmonary hypertension. Given their non-invasive and safe nature, ECG, chest X-ray, and echocardiography are frequently employed in combination for HAPH screening.^73^ In addition, PFT is primarily utilized to exclude chronic obstructive pulmonary disease (COPD) and interstitial lung disease (ILD).^89,90^ Right heart catheterization remains the gold standard diagnostic approach for HAPH, with mPAP > 30 or sPAP > 50 mm Hg, which differs from other types of pulmonary hypertension that are characterized by an mPAP > 20 mm Hg.^15^ HAPH diagnosis requires the exclusion of secondary causes (left heart disease, thromboembolism, etc.).^90^ HAPH pathology primarily involves the pulmonary vasculature and right heart.^68^ Cardiac changes include biventricular hypertrophy (RV comprising 67% of heart weight compared with 30% of normal weight), myofiber degeneration, calcification, and mitochondrial damage. Pulmonary arterioles exhibit medial thickening and muscularization of small arteries (<100 μm), driven by smooth muscle cells (SMCs) proliferation, intimal hyperplasia, and adventitial fibrosis. Endothelial swelling narrows/obstructs lumens, causing widespread pulmonary arterial thrombosis. Pulmonary vascular lesions feature medial hypertrophy, muscularization of small arterioles, intimal hyperplasia, and endothelial swelling causing luminal occlusion. Thrombosis frequently occurs in medium/small arteries.

### Genetic susceptibility in altitude illness pathogenesis

Susceptibility to altitude illnesses varies markedly among individuals and populations, with an emerging consensus that genetic mechanisms underlie this phenomenon. Advances in genomic sequencing have substantially supported the role of genetic predisposition in altitude illness (Table 1). While understanding the genetic basis of altitude-related illnesses holds promise for improving the prevention, diagnosis, and treatment, the current knowledge of how genetic variations influence susceptibility remains limited. Therefore, broader data collection is essential to elucidate the repeatability/stability of the genetic basis of altitude illnesses across different biogeographical groups, which in turn will facilitate clinical translation.

**Table 1:** Genetic predispositions for high altitude illness

Gene	Full name and function	SNPs	Altitude illness	Population	Reference
*EPAS1*	Endothelial PAS domain-containing protein 1; associated with mild gastrointestinal symptoms.	rs6756667	AMS	320 AMS and 284 control, Chinese Han men	^95^
	Correlated with the SaO_2_ level and AMS susceptibility.	rs4953348	AMS	95 AMS and 176 control, Chinese Han men	^94^
	Associated with elevated AMS risk.	rs6756667, rs13419896,rs4953354	AMS	369 AMS and 234 control, Chinese Han men	^93^
*EGLN1*	EGL nine homolog 1; associated with elevated AMS risk.	rs2153364	AMS	320 AMS and 284 control, Chinese Han men	^95^
	Associated with AMS susceptibility.	rs12406290,rs2153364	AMS	190 AMS and 190 control, Chinese Han men	^97^
*VEGFA*	Vascular Endothelial Growth Factor A; related to mild headache.	rs3025029	AMS	320 AMS and 284 control, Chinese Han men	^95^
	Associated with a decreased risk of AMS.	rs3025039, rs3025030	AMS	200 AMS and 200 control, Chinese Han men	^96^
*HIF1AN*	Hypoxia-inducible factor 1-alpha inhibitor; associated with increased risk of AMS.	rs12406290,rs2153364	AMS	190 AMS and 190 control, Chinese Han men	^97^
*FAM149A*	*Homo sapiens* family with sequence similarity 149 member A; associated with AMS susceptibility.	4 SNPs in the FAM149A gen	AMS	43 AMS and 56 control, Nepal	^100^
*NOS3*	Nitric oxide synthase 3; associated with susceptibility to AMS.	rs1799983	AMS	33 AMS and 59 control, Nepal	^101^
*GSTM1*	Glutathione S-transferase M1; associated with AMS susceptibility.	rs366631	AMS	43 AMS and 80 control, Chinese soldiers	^102^
*GSTT1*	Glutathione S-transferase theta 1; associated with AMS susceptibility.	rs2266637	AMS	43 AMS and 80 control, Chinese soldiers	^102^
*PPARA*	Peroxisome Proliferator-Activated Receptor Alpha; associated with elevated AMS risk.	rs7292407	AMS	320 AMS and 284 control, Chinese Han men	^95^
*ACE*	Angiotensin-Converting Enzyme; associated with HAPE.	rs4343	HAPE	140 HAPE and 144 Control, Chinese Han men	^111^
		rs8066114, rs4461142		27 HAPE and 108 control, Chinese Han	^112^
		rs1799752		64 HAPE patient and 53 HAPE resistant control, India	^116^
*CYP11B2*	Cytochrome P450 Family 11 Subfamily B Member 2; associated with HAPE.	rs4149178, rs1799998	HAPE	140 HAPE and 144 Control, Chinese Han men	^111^
*NOS3*	Nitric oxide synthase 3; associated with increased HAPE risk.	rs1799983	HAPE	27 HAPE and 108 control, Chinese Han	^112^
*EGLN1*	EGL nine homolog 1; found crucial for adaptation and maladaptation in the highland (3500 m) Ladakh population.	rs1538664, rs479200, rs2486729, rs2790879, rs480902, rs2486736, rs973252	HAPE	250 HAPE patient, 210 HAPE-free (India) and 430 Healthy Ladakhi control (Ladakhi)	^118^
	Associated with increased HAPE risk.	rs479200, rs480902	HAPE	96 HAPE and 96 control, India	^117^
*AGT*	Angiotensinogen; found a significant association of M allele of AGT with HAPE.	rs4762	HAPE	160 HAPE patient and 163 HAPE resistant control, India	^114^
*ADRB2*	Adrenoceptor Beta 2; associated with HAPE were the haplotypes from 46 A/G and 79 C/G SNP of ADRB2.	rs1042713, rs1042714, rs1042711	HAPE	110 HAPE patient and 143 HAPE resistant control, India men	^125^
*TH*	Tyrosine hydroxylase; may not be sufficient as a genetic marker for predicting a predisposition to HAPE.	rs1799931	HAPE	43 HAPE and 51 control, India men	^116^
*ET-1*	Endothelin-1; associated with HAPE.	rs5370	HAPE	64 HAPE patient and 53 HAPE resistant control, India	^116^
*APLN*	Apelin; found between HAPE susceptibility and variants of APLN were putative associations.	rs3761581, rs2235312, rs3115757	HAPE	96 HAPE patient,96 HAPE-free and 96 HLs, India men	^123^
*APLNR*	Apelin receptor; found between HAPE susceptibility and variants of APLNR were putative associations.	rs11544374,rs2282623	HAPE	96 HAPE patient,96 HAPE-free and 96 HLs, India men	^123^
*CYBA*	Cytochrome b-245 alpha chain; did not play a substantial role in the pathogenesis of HAPE.	rs4673	HAPE	39 HAPE and 43 control, European Caucasians	^126^
*GSTP1*	Glutathione S-Transferase Pi 1; associated with elevated circulating 8-iso-prostaglandin F2α in HAPE.	rs1695, rs1138272	HAPE	150 HAPE patients, 180 HAPE resistant control and 180 HLs, India	^126^
*EPAS1*	Endothelial PAS domain-containing protein 1; associated with high altitude adaptation in the Tibetans.	rs2305389	HAPE	153 HAPE patient, 298 HAPE resistant healthy controls(Chinese Han) and 245 healthy highland Tibetans(Chinese Tibetans)	^127^
*TIMP3*	Tissue Inhibitor of Metalloproteinases 3; the minor allele C of rs130293 (C/T) in the TIMP3 gene was linked to resistance to HAPE, while the ancestral allele T was associated with susceptibility to HAPE.	rs130293	HAPE	53 subjects susceptible to HAPE and 67 resistant to HAPE, Japan	^129^
*HSPA1A*	Heat Shock Protein Family A (Hsp70) Member 1A; associated with the susceptibility to HAPE.	rs1043618, rs1008438	HAPE	148 HAPE and 483 control, Chinese Han men	^128^
*HSPA1B*	Heat Shock Protein Family A (Hsp70) Member 1B; associated with the susceptibility to HAPE.	rs1061581	HAPE	148 HAPE and 483 control, Chinese Han men	^128^
*SFTPA1*	Surfactant Protein A1; associated with the susceptibility to HAPE.	rs1130142, rs713323, rs1130143	HAPE	12 HAPE, 15 healthy LAN sojourners control and 19 healthy high-altitude natives control	^130^
*SFTPA2*	Surfactant Protein A2; associated with the susceptibility to HAPE.	rs1130144	HAPE	12 HAPE, 15 healthy LAN sojourners control and 19 healthy high-altitude natives control	^130^
*SENP1*	SUMO-specific protease 1; provided independent evidence in support of a role for SENP1 in CMS in individuals of Quechua ancestry.	rs7963934	CMS	84 CMS and 91control, Peru	^134^
	Associated with higher CMS scores were non-G/G genotypes.	rs7963934	CMS	71 CMS and 110 control, Peru	^135^
*ACE*	Angiotensin converting enzyme I/D; associated with CMS.	rs4340	CMS	50 CMS and 36 control, Chinese Tibetans	^136^
*AGT*	Angiotensinogen; associated with CMS.	rs699	CMS	50 CMS and 36 control, Chinese Tibetans	^136^
*VEGFA*	Vascular Endothelial Growth Factor A; associated with CMS.	rs3025033	CMS	131 CMS and 84 control, Peru	^137^
*ACE*	Angiotensin-Converting Enzyme I/D; provided the first example of a gene conferring susceptibility to HAPH.	rs1799752	HAPH	22 HAPH and 15 control, Kyrgyz men	^138^
	Associated with HAPH.	rs1799752	HAPH	48 HAPH and 30 control, Kyrgyz men	^71^

#### Acute altitude illness

##### AMS and HACE

AMS diagnosis relies on subjective composite symptom scoring and complex pathophysiology, resulting in heterogeneous genetic datasets. Thus, even among populations with similar backgrounds, the cohorts showed significant genetic differences (Table 1). Notably, variants in *EPAS1*, *EGLN1*, and *VEGFA* were consistently associated with AMS susceptibility across several investigations.^92^ Four single nucleotide polymorphisms (SNPs) (rs13419896, rs4953348, rs4953354, and rs6756667) were found to be associated with an elevated risk of AMS development, especially *EPAS1* rs6756667, which has been consistently validated by two independent cohorts and further identified as being associated with AMS-related mild gastrointestinal symptoms.^93–95^ The *VEGF* SNPs rs3025030 and rs3025039 have been implicated in the risk of developing AMS, and rs3025039 has been linked to AMS-related mild headaches.^95,96^ The *EGLN1* “GG” haplotype (rs12406290/rs2153364) increased AMS risk,^97^ with rs2153364 validated by Huang’s group.^95^ Hypoxia response genes (hypoxia-inducible factor (*HIF*)*1* *A*, *HIF1AN*, and *VHL*) showed no significant association with AMS in either the Chinese Han or Sherpa populations.^97–99^

MacInnis et al. reported that 4 *FAM149A* SNPs were associated with AMS using genome-wide association study (GWAS), although these findings were not unreplicated in another cohort, suggesting possible false positives or small effects.^100^ *NOS3* variants, which are involved in NO synthesis, showed controversial AMS associations in Chinese (no association, n = 128) and Nepal (association, n = 92) populations.^98,101^ Additionally, *PPARA*, *GSTM1* and *GSTT1* also contributed to AMS susceptibility.^95,102^ However, genetic variants in *ADRB2*^103^ and *ACE*^104–107^ (four separate studies with 103–284 subjects) did not appear to be linked with AMS development or symptoms. While candidate genes are identified, their generalizability and clinical utility require validation.

Currently, since HACE is a rare form of altitude encephalopathy, no studies have explored its potential genetic basis. Although HACE is widely regarded as a severe progression of AMS,^108^ whether HACE and AMS share common genetic susceptibility factors remains unknown and requires dedicated investigation.

##### HAPE

The clearer diagnosis of HAPE makes it more suitable for genetic studies than AMS or HACE (Table 1). The role of *ACE* genetic variants in HAPE development has been a subject of controversy. Early small studies (n = 39–104, HAPE cases<50) found no association between *ACE I/D* polymorphism and HAPE susceptibility,^107,109,110^ whereas larger studies (n = 117–323, HAPE > 100) reported a significant link between certain *ACE* SNPs (*ACE I/D*, rs4309, rs4343, rs4461142, and rs8066114) and the risk of developing HAPE.^111–116^ Critically, multiple cohorts consistently identified the significant association between *ACE I/D* polymorphism and HAPE susceptibility.^114–116^ The conflicting conclusions may be attributed to the diverse sample sizes utilized across the studies, thus warranting more comprehensive investigations in large population cohorts using advanced genome-wide techniques.

Conversely, a consistent correlation between *EGLN1* SNPs and HAPE susceptibility has been observed in multiple independent studies. In a GWAS conducted by Aggarwal et al., *EGLN1* rs479200 and rs480902 were associated with higher expression of *EGLN1* and were more prevalent in HAPE patients compared to native highlanders from Indian populations.^117^ This finding was further confirmed by Mishra et al. in a larger Ladakhi cohort, which revealed that seven *EGLN1* polymorphisms (rs1538664, rs479200, rs2486729, rs2790879, rs480902, rs2486736 and rs973252) were strongly correlated with HAPE susceptibility.^118^ These susceptible genotypes were further identified to be linked with increased *EGLN1* expression and decreased SaO_2_. Furthermore, a study among Han recruits also revealed a significant correlation between *EGLN1* rs480902 and increased risk of HAPE.^113^ The consistent identification of *EGLN1* rs480902 across different biogeographical groups underscores its potential role in assessing HAPE risk. However, the predictive diagnostics and functional validations need to be further addressed.

The association between *NOS3* genetic variants and HAPE susceptibility appears to be complex and varies across different geographical and ethnic populations. While Johanna et al. found no significant association between *NOS3* rs1799983 polymorphism and HAPE susceptibility in Caucasians,^119^ multiple studies in different geographical populations, including Japanese,^120^ Chinese,^112,113^ and Indian^121–123^ populations, have highlighted a robust correlation between *NOS3* rs1799983, rs199983, and rs7830 polymorphisms and HAPE risk. Particularly, the rs1799983 polymorphism, which is associated with the reduced NO level in HAPE patients, emerged as one of the most extensively studied and validated genetic markers for HAPE susceptibility.^112,120–123^ Notably, a meta-analysis (n = 399 HAPE/495 controls) confirmed the significance of the rs1799983 polymorphism in increasing HAPE risk among Asians.^112^ These findings underscore the importance of *NOS3* rs1799983 polymorphism as a candidate biomarker for HAPE susceptibility. Further functional studies and clinical applications of this polymorphism could significantly contribute to reducing HAPE incidence and improving preventive and therapeutic strategies.

Genetic variations in vascular homeostasis-related genes, particularly those related to the renin‒angiotensin‒aldosterone system (RAAS) and adrenergic signaling, have been implicated in HAPE pathogenesis. The *AGT* rs699 and rs4762 polymorphisms have been found to be significantly associated with HAPE susceptibility in Chinese and Indian populations, respectively.^114,124^ Two independent studies have validated the correlation between *CYP11B2* rs4149178 and the risk of developing HAPE.^109,124^ Genetic variations in *ADRB2*, particularly the rs1042713 and rs1042714 polymorphisms, were associated with increased susceptibility to HAPE, potentially due to their effects on lung fluid accumulation.^125^ A genome scan revealed that genetic variations in *apelin* (rs3761581/rs2235312/rs3115757) and its receptor *APLNR* (rs11544374/rs2282623) were significantly associated with HAPE.^123^ They further identified that the risk alleles, rs3761581G and rs2235312T were related to lower levels of *apelin* expression and nitrite, highlighting the complex interplay of genetic factors in HAPE pathogenesis. The *ET-1* rs5370 polymorphism was also implicated in HAPE susceptibility,^123^ but two tyrosine hydroxylase (*TH*) polymorphisms showed no relationship.^116^

Additional genes, such as *CYBA*, *GSTP1*, *EPAS1*, *HSPA1A*, *HSPA1B*, *TIMP3*, *SFTPA1*, and *SFTPA2*, have also been implicated in HAPE development. An initial study with a limited sample size (39 HAPE patients and 43 controls) revealed no role of CYBA in HAPE pathogenesis in Caucasians,^119^ but a larger Indian cohort (150 HAPE patients and 180 controls) linked *CYBA* (rs4673 and rs9932581) and *GSTP1* (rs1695 and rs1138272) to elevated circulating 8-iso-prostaglandin F2α in HAPE, potentially contributing to HAPE susceptibility.^126^ For genes in the hypoxia response pathway, Ge’s group revealed that the *EPAS1* rs2305389 was strongly related to HAPE risk among Han Chinese.^127^ Regarding heat shock protein genes, *HSPA1A* rs1043618/rs1008438 and *HSPA1B* rs1061581 have also shown significant correlation with HAPE susceptibility in Qinghai‒Tibet railway workers.^128^ Besides, the minor allele C of *TIMP3* rs130293 was associated with HAPE resistance, whereas the ancestral allele T was linked to susceptibility, which requires further validation in diverse populations.^129^ Polymorphisms in *SFTPA1* (rs1130142, rs713323, and rs1130143) and *SFTPA2* (rs1130144) were suggested as possible genetic factors contributing to HAPE susceptibility, but the small sample size (12 HAPE patients and 15 controls) of the study limits its reliability.^130^

In conclusion, over the years, numerous studies have explored the role of genetic variations in HAPE susceptibility, with investigations spanning diverse sample sizes and population backgrounds. Despite the variability, several consistent findings have emerged. It is evident that HAPE is not caused by a single gene but rather by a complex and dynamic network of genes. The interplay between different genetic variants, along with environmental factors, contributes to HAPE susceptibility. Thus, future research should focus on developing diagnostic models based on genetic variations to predict HAPE susceptibility as accurately as possible. Additional studies are needed to further elucidate the precise mechanisms by which these genetic variants disrupt physiological processes, ultimately leading to HAPE development.

#### Chronic altitude illness

##### CMS

Unlike acute altitude illnesses, research on the genetic basis of CMS is limited, with only a few studies exploring the link between CMS susceptibility and several genes, including *SENP1*, *ACE*, *AGT*, and *VEGFA* (Table 1). Additionally, these findings have yet to be consistently replicated across independent cohorts. Previous epidemiological studies have indicated significant differences in CMS prevalence among different ethnic groups, with a notably lower prevalence in Tibetan highlanders compared to Andean highlanders and Han Chinese.^131,132^ Specific SNPs in *EPAS1*, *EGLN1*, and *PPARA* genes are closely associated with low Hb concentrations, suggesting a genetic basis for reduced erythropoietic response and protection against CMS. Unexpectedly, SNPs related to HIF/EPO pathway genes (*EGLN1*, *EGLN2*, *EGLN3*, *EPO*, *EPOR*, *HIF1A*, *PTEN*, and *VHL*) have failed to establish a definitive correlation with CMS susceptibility among male Peruvian Quechua natives, potentially due to flawed grouping: non-CMS controls lacked strict criteria (non-CMS: individuals with a CMS score < 12 or an Hb concentration < 213 g/L).^133^ Thus, further investigation is warranted to elucidate whether genetic variations in genes related to the HIF/EPO pathway contribute to CMS pathogenesis.

Recent findings have shifted the focus toward *SENP1*, not the classic HIF-regulated genes, as a potential differentiator between healthy Andean highlanders and CMS patients. Cole and colleagues reported a significant association between *SENP1* rs7963934 polymorphism and CMS susceptibility, possibly through its role in modulating erythropoiesis.^134^ A study (110 healthy controls and 71 CMS patients) conducted by Hsieh et al. validated this finding and linked G/G genotype of *SENP1* rs7963934 to lower Hb levels and CMS scores.^135^ Besides, Norman et al. reported that *ACE I/D* (rs4340) and *AGT* rs699 were significantly related to CMS susceptibility in Tibetan populations, with rs4340 correlating with heart rate.^136^ The AG genotype of *VEGFA* rs3025033 was found to confer a 2.5-fold increased risk of CMS compared to GG genotype in another Andean cohort.^137^

Research on genetic variations associated with CMS susceptibility faces several challenges, including a limited number of study cohorts, small sample sizes, and complex diagnostic criteria, yielding heterogeneous genetic data. Future research should aim to identify novel genetic variation and replicate these findings in larger, more diverse populations by incorporating multiomics approaches, such as genomics, transcriptomics, and proteomics, to comprehensively explore the genetic and molecular mechanisms involved in CMS.

##### HAPH

In contrast to other altitude-related diseases, research into the genetic mechanisms underlying HAPH development is still in its infancy. The few existing studies, characterized by extremely small sample sizes, have identified only a small number of genes associated with HAPH (Table 1). Studies by Morrell et al.^138^ (22 HAPH patients and 15 controls) and Aldashev et al.^71^ (48 HAPH patients and 30 controls) in Kyrgyz populations revealed a greater frequency of the *ACE I/I* genotype among individuals with HAPH compared to controls. This is paradoxical since the I allele typically reduces ACE activity to reduce the availability of angiotensin II, thereby promoting vasodilation. The observed association suggested a more nuanced role of *ACE* in HAPH pathogenesis, potentially enhancing endurance performance to augment cardiac output and PAP. Alternatively, the *ACE I/D* polymorphism may serve as a genetic marker for HAPH susceptibility, independent of its effects on ACE activity in other systems. More recently, Iranmehr and colleagues conducted a whole genomic sequence among 18 Kyrgyz subjects (9 HAPH patients and 9 controls) to identify additional candidate genes, including *MTMR4*, *TMOD3*, and *VCAM1*, which are functionally associated with well-known molecular and pathophysiological processes of pulmonary hypertension.^139^ However, the limited sample size may introduce potential biases, resulting in restricted analysis of genetic variants involved in HAPH pathogenesis. Overall, the genetic architecture of HAPH remains largely unknown due to the limited number of studies and small sample sizes. Therefore, larger and more comprehensive studies are needed to confirm these preliminary observations and identify additional genetic markers for HAPH susceptibility.

### Pathogenic mechanisms of AMS and HACE

**Fig. 2:**
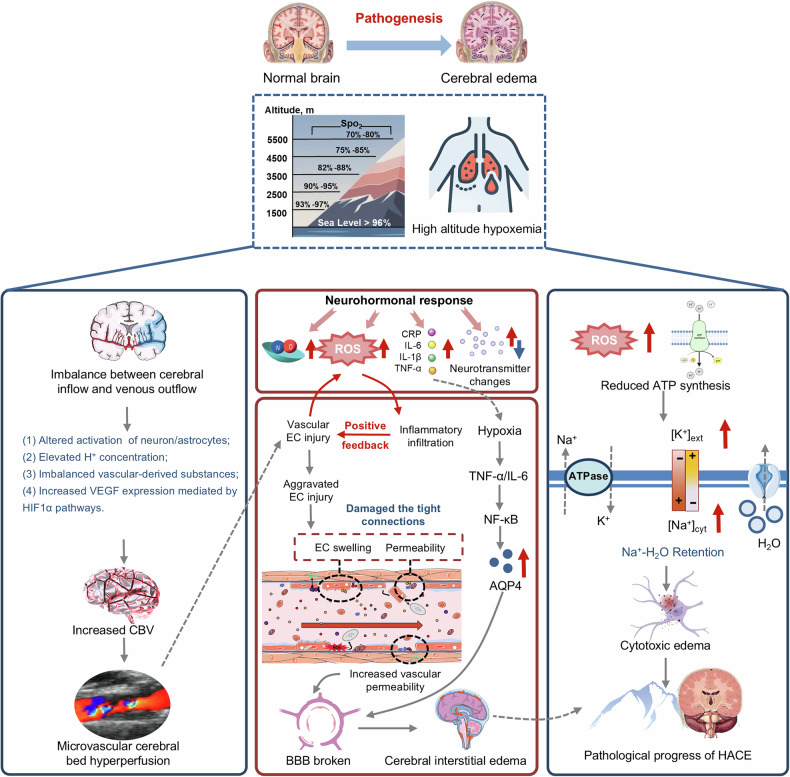
Pathophysiology of AMS and HACE. There are three interconnected mechanisms involved in HACE progression under altitude hypoxia: **a** cerebral hemodynamic imbalance due to disrupted arterial inflow and venous outflow, driven by neurovascular unit dysfunction, elevated [H⁺], vasoactive mediators, and HIF-1α-mediated VEGF upregulation, leading to increased CBV and microvascular hyperperfusion; **b** neurohormonal and inflammatory responses involving ROS, proinflammatory cytokines (e.g., CRP, IL-6, TNF-α), and neurotransmitter alterations, which induce endothelial cell injury, tight junction disruption, and BBB breakdown, resulting in vasogenic edema; and **c** the cytotoxic edema cascade initiated by impaired Na⁺/K⁺-ATPase due to reduced ATP synthesis, causing intracellular Na⁺ and water accumulation

AMS and HACE are often considered a pathophysiological continuum of cerebral high-altitude illness with neurological dysfunction. It is generally accepted that if left untreated, severe AMS usually progresses to HACE, suggesting that both diseases may share an initial common pathophysiology.^108^ While hypoxia is the primary trigger, the underlying mechanisms leading to clinical symptoms require elucidation. Furthermore, whether HACE is a severe form of AMS or an independent disease remains elusive, and the triggers and mechanisms for the progression from severe AMS to HACE remain incompletely understood.^8,108^ An increasing number of studies have elucidated key mechanisms contributing to AMS and HACE pathogenesis (Fig. 2). In summary, altitude hypoxemia caused by hypobaric hypoxia elicits neuro-hormonal responses, encompassing alterations in neurotransmitters, reactive oxygen species (ROS), cytokines (especially inflammatory cytokines), nitric oxide (NO), and eicosanoids among others, and cerebral hemodynamic disorders (increased cerebral blood velocity (CBV) resulting from an imbalance between arterial inflow [cerebral blood flow (CBF)] and venous outflow). Subsequently, hyperperfusion and inflammation occur in microvascular cerebral beds to disrupt the tight junctions between endothelial cells of cerebral arteries by elevating mechanical pressure, increasing cerebral vascular permeability, and swelling endothelial cells. Consequently, the damaged blood‒brain barrier (BBB) permits capillary leakage, leading to cerebral vasogenic edema. Besides, hypoxia-induced excessive ROS production and reduced ATP synthesis impair Na^+^/K^+^ ATPase pump, resulting in cytotoxic edema. Both cerebral vasogenic and cytotoxic edema contribute to HACE pathogenesis, although a comprehensive understanding of HACE pathogenesis is lacking.

#### Neuro-hormonal responses: neurotransmitters, ROS, and inflammatory cytokines

Animal studies suggest that altered neurotransmitter release contributes to brain dysfunction under hypoxia. Decreased synthesis of acetylcholine, which is closely related to oxidative metabolism, may contribute to the fatigue common at high altitude.^140^ Moreover, in vivo synthesis of serotonin (5-HT) decreases with oxygen deprivation, suggesting an abnormal 5-HT-mediated function in the development of hypoxia-induced AMS and HACE symptoms.^141,142^ Thus, further studies are required to determine the precise role of hypoxia-influenced neurotransmitter alterations in the pathogenesis of cerebral high-altitude illness. Except neurotransmitter systems, several biohumoral factors, mostly HIF-dependent, participate in a variety of pathophysiological processes of high-altitude diseases, including ROS, cytokines (especially inflammatory cytokines), NO, and eicosanoids.^143,144^ Notably, cohort studies have linked genetic variations in HIF pathway-related genes (*EPAS1*, *EGLN1*, *PPARA*, *GSTM1*, and *GSTT1*) to AMS/HACE prevalence and/or severity (Table 1).

ROS, comprising molecules derived from molecular oxygen (such as superoxide (O₂•⁻), hydrogen peroxide (H_2_O_2_), hypochlorous acid (HOCl), hydroxyl (•OH), alkoxyl (RO•), and peroxyl (ROO•) radicals), are generated mainly by mitochondria.^145^ Under hypoxia, ROS production increases significantly via various mechanisms.^146^ Excessive ROS generation causes oxidative stress, resulting in DNA breakage, protein denaturation and aggregation, mitochondrial dysfunction, lipid peroxidation, and altered cell membranes, ultimately promoting cell death.^145,147^ Evidence also supports the role of elevated ROS in AMS and HACE pathogenesis. Multiple studies have demonstrated that excessive oxidative stress is a pathogenic factor for AMS and HACE development, which is supported by increased oxidative stress markers.^148–154^ A study in humans indicated that increased cerebral oxidative‒nitrative stress during hypoxia was positively correlated with AMS/headache scores, independent of BBB disruption (Fig. 2, right).^155^ Irarrázaval et al. demonstrated that the oxidative stress markers were upregulated in volunteers exposed to acute hypoxia, with plasma lipid peroxidation significantly correlated with AMS severity (Fig. 2, right).^156^ Moreover, free radicals (a subtype of ROS) were upregulated and closely related to BBB impairment, suggesting a potential contribution to HACE development (Fig. 2, right).^157,158^

Genetic and proteomic studies suggest that inflammation also contributes to AMS and HACE by impairing the BBB. After acute hypoxic exposure, the levels of inflammatory cytokines, including C-reactive protein (CRP), IL-1β IL-6, IL-17RA, and TNF-α, were obviously higher in AMS-susceptible individuals than AMS-resistant, whereas the anti-inflammatory cytokine IL-10 was lower (Fig. 2, middle).^159–161^ Furthermore, multiple studies revealed that elevated TNF-α, IL-1β, and IL-6 or reduced IL-10 were positively associated with AMS severity, implicating dysregulated inflammation in AMS pathogenesis (Fig. 2, middle).^162–164^ Animal experiments also showed upregulation of inflammatory cytokines (TNF-α, IL-1β, and IL-6) in mice or rats acutely exposed to hypoxia (Fig. 2, middle).^164,165^ Mechanistically, microglia might be activated by altitude hypoxia and migrate to cerebral microvessels.^166^ Cytokines (TNF-α and IL-6), released by activated microglia, promoted astrocyte edema through activating the TLR4-mediated MAPK and NF-κB signaling pathways in astrocytes, thereby upregulating aquaporin 4 (AQP4) (Fig. 2, middle).^167,168^ Increased AQP4 expression, the major water channel facilitating water influx into astrocytic end-feet and across the BBB, enhanced water permeability, causing astrocyte swelling, tissue damage, and exacerbated edema.^169^ These pathological changes disrupted the BBB, eventually contributing to HACE. Additionally, bradykinin, histamine, arachidonic acid, and NO may also alter BBB function, involved in HACE occurrence.^158^

#### Cerebral hemodynamics and rheological changes

During ascent to a high-altitude area, hypoxemia causes a marked increase in CBF, resulting in significant increases in CBV and blood pressure, which may contribute to headache and neurological dysfunctions.^170^ Elevated intracranial pressure (ICP) and/or release of nociceptive mediators contribute to capillary leakage and hemorrhages, potentially leading to vasogenic edema.^171^ CBF increases promptly upon ascent to high altitude and slowly returns to normal over 1-3 weeks.^172,173^ Moreover, the magnitude of CBF is positively related to attained altitude, a dose-dependent effect confirmed by multiple studies; however, the role of ascent rate in CBF response remains unclear.^173,174^ Hypoxic cerebrovascular reactivity studies indicated that CBF increased by approximately 0.5% to 2.5% for each 1% decrease in arterial saturation of O_2_ (SaO_2_).^175–181^ Several mechanisms were involved in hypoxaemia-induced evelation in CBF (Fig. 2, left), encompassing (1) when oxygen availability was lowered, the altered activity of neurons/astrocytes in the neurovascular unit facilitated vasodilation^182,183^; (2) upon reduction in PaO_2_, the elevated H^+^ concentration, due to excessive lactate formation by euronal/glial anaerobic metabolism, might be responsible for cerebral vasodilatation^184^; and (3) a myriad of vascular-derived substances (adrenaline, adenosine, angiotension-II, NO, prostaglandins (PGE), and endothelium-derived hyperpolarizing factor (EDHF)) also contributed to vasodilatation and an increase in CBF during exposure to hypoxia.^185^ However, the specific mechanisms regulating CBF via these processes remain incompletely understood, and the roles of NO and adenosine in hypoxic cerebrovascular dilatation remain uncertain.^172,186^

There is a long-standing debate concerning the contribution of marked increases in CBF to AMS and HACE pathogenesis. A study found higher CBF velocity in subjects with AMS than in those without, directly associated with AMS severity.^187^ However, later studies did not confirm this finding.^174,188,189^ Nevertheless, given other risk factors (such as genetic profile, sleep, exercise) and the complexity of regional CBF regulation (previous studies primarily assessed global CBF), insufficient data exist to define the role of increased CBF in AMS /HACE development. Notably, recent landmark studies proposed that an imbalance between cerebral inflow (i. e. CBF) and venous outflow was crucial in AMS and HACE pathophysiology (Fig. 2, left). Following hypoxia-associated CBF elevation, which resulted in cytotoxic and vasogenic edema, as evidenced by increase in gray and white matter volumes, anatomical restrictions in cerebral venous outflow might lead to venous engorgement and a subsequent rise in ICP, contributing to headache burden at high altitudes.^190,191^ Normobaric hypoxia studies further revealed that ICP alterations were significantly associated with AMS symptom severity, implicating venous outflow restriction as a key mechanism in AMS and HACE development.^191,192^ Therefore, both increased CBF (contributing to cytotoxic and vasogenic edema) and restricted venous outflow are involved in AMS and HACE development.

Except increased ICP resulting from altered hemodynamics under hypoxia, elevated vascular endothelial growth factor (VEGF) may also contribute to AMS and HACE pathogenesis via promoting vascular permeability and impairing BBB integrity. Importantly, VEGF variants are associated with elevated AMS risk, where *VEGF* rs3025039 is associated with AMS-related mild headaches. Several reports indicate that plasma VEGF increases upon ascent to high altitude, but found no correlation with AMS (Fig. 2, left; Table 1).^193–195^ Tissot van Patot et al. reported that soluble VEGF receptor (sFlt-1), which binds circulating VEGF to reduce its bioavailability, was lower in subjects with AMS than without, and lower sFlt-1 levels were correlated with AMS severity, indicating that functional VEGF levels are essential in AMS pathology.^196^ However, further prospective study revealed increased levels of VEGF and sFlt-1 after ascent, but neither was correlated with AMS symptoms.^197^ These findings do not exclude the role of VEGF in AMS and HACE, since plasma measurements may not reflect the effects of paracrine intracerebral VEGF. Animal experiments strongly supported VEGF-mediated cerebral vasogenic edema in AMS and HACE pathogenesis. VEGF mRNA and protein levels were significantly upregulated in mice and rats exposed to 6–10% O_2_ (corresponding to an altitude of 4000–9500 m) (Fig. 2, left).^198,199^ Increased vascular permeability in mice exposed to 8% oxygen could be completely prevented by VEGF neutralizing antibodies, suggesting that hypoxia-induced VEGF production causes brain vasogenic edema (Fig. 2, left).^198^ These findings demonstrate that VEGF antagonists may be promising agents for preventing and treating AMS and HACE.

Besides, cerebrovascular autoregulation modulated CBF to accommodate arterial perfusion changes, protecting the brain against hypoxic challenge.^200^ With hypoxia at 15% O_2_, autoregulation became dysfunctional, causing BBB disruption and vasogenic cerebral edema.^201,202^ However, the role of hypoxia-mediated autoregulation impairment in AMS remains controversial.^203,204^

#### Fluid alteration

Owing to small sample sizes and contradictory results in most studies, the role of fluid retention in AMS development remains unclear. Compared with those without AMS, subjects with AMS exhibit greater early water retention due to lower fluid loss than those without, indicating a link with AMS symptoms.^205,206^ However, recent studies showed that fluid retention was not essential for AMS pathogenesis, as total body water (TBW) was similar between groups with and without AMS.^207,208^ The mechanisms underlying the putative role of fluid retention in AMS are also unclear. Hackett et al. proposed that hypoxemia-induced activation of peripheral chemoreceptors might increase extracellular water by upregulating circulating antidiuretic hormone (ADH) levels, thus promoting BBB permeability and increasing the ICP to cause AMS symptoms.^209^ Other studies also suggested that acute hypoxia elevated circulating ADH and aldosterone^205,210^ and decreased atrial natriuretic peptide (ANP),^211^ potentially contributing to anti-diuresis and fluid retention. Moreover, Jack et al. revealed a direct relationship between elevated ADH and AMS severity.^205^

In contrast, evidence supported the potential involvement of reduced circulating volume (fluid loss) in AMS pathogenesis, challenging the typical hypothesis proposed by Hackett’s group in 2001. Multiple groups demonstrated that individuals, exposed to high altitude exhibited increased ANP, brain natriuretic peptide (BNP), epinephrine, endothelin-1 (ET-1), and adrenomedullin levels, as well as decreased ADH, renin, and aldosterone levels, leading to hypoxic a diuretic response.^205,211–215^ Furthermore, elevated BNP was associated with increased AMS occurrence and severity.^214–216^ However, the correlation between the above altered ANP/BNP/ADH levels and TBW needs additional investigations. Notably, the ANP level was not significantly different between those with and without AMS.^211^ In conclusion, further studies are needed to address the mechanisms of hypoxia-mediated fluid imbalance and determine whether and how fluid retention/loss contributes to AMS/ HACE development.

### Pathogenic mechanisms of HAPE

**Fig. 3:**
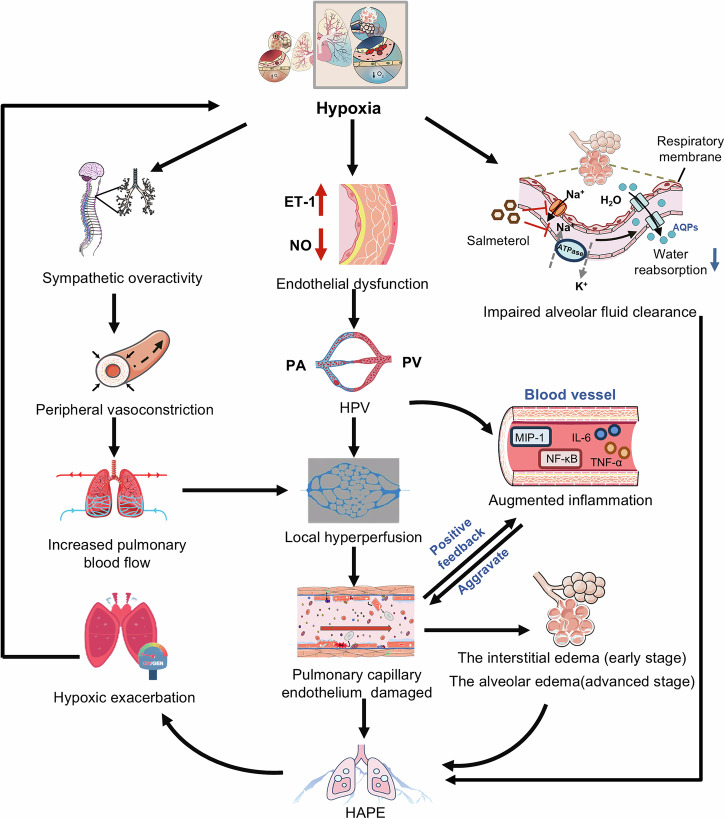
Pathophysiology of HAPE. Hypoxia simultaneously induces sympathetic overactivity (leading to increased pulmonary blood flow) and endothelial dysfunction (characterized by elevated [ET-1] and reduced [NO], driving hypoxic pulmonary vasoconstriction [HPV] and local hyperperfusion). These combined hemodynamic alterations cause local hyperperfusion, which, along with impaired alveolar fluid clearance (due to defective Na⁺/water transport across the respiratory membrane) and augmented inflammation (e.g., MIP-1, IL-6, and TNF-α), synergistically cause pulmonary capillary endothelial damage. This pathology progresses to interstitial edema (early-stage HAPE) and alveolar edema (advanced-stage HAPE)

Hypoxia is indeed the initiating factor that triggers a series of physiological responses ultimately culminating in the development of HAPE.^217^ Mounting evidence has revealed the potential mechanisms involved in HAPE pathogenesis (Fig. 3). In summary, HAPE results from a persistent imbalance between the forces that drive the accumulation of fluid within the airspace and the biological processes responsible for its elimination. The amount of fluid that leaks into the airspace from high-permeability pulmonary vasculature is directly associated with the degree of hypoxic pulmonary hypertension, caused by an exaggerated hypoxic pulmonary vasoconstriction (HPV). Mechanistically, exaggerated hypoxic pulmonary hypertension is related primarily to defective pulmonary NO synthesis, increased ET-1 synthesis, and enhanced sympathetic activation. It is conceivable that both regional overperfusion and elevated vascular resistance, caused by inhomogeneous HPV and hypoxic venoconstriction, respectively, contribute to increased pulmonary capillary transmural pressures, which leads to stress failure of pulmonary capillaries to aggravate vascular permeability. Additionally, inflammation appears to be a secondary response to alveolar edema and microvascular disruption, which in turn aggravates pulmonary edema via enhancing pulmonary capillary permeability. The impaired alveolar fluid clearance, identified by Urs and colleagues, is largely dependent on defective respiratory transepithelial sodium and water transport. Consequently, the resultant alveolar flooding caused by hypobaric hypoxia leads to progressive hypoxemia and even death if untreated.

#### Exacerbated pulmonary hypertension

Exacerbated hypoxic pulmonary hypertension is a hallmark of HAPE. After arriving at high altitude, PAP usually increases, which can be alleviated by oxygen administration. However, individuals who are prone to HAPE exhibit an abnormal increase in PAP in response to acute hypoxia. The precise etiology underlying accentuated PAP remains to be fully elucidated and is likely multifactorial, encompassing sympathetic overactivation and endothelial dysfunction (such as decreased availability of NO and elevated levels of ET-1). Over the years, numerous cohorts have identified the significant associations between genetic variations in vascular homeostasis-related genes (*ACE*, *AGT*, *AGTR1*, *NOS3*, *Apelin*, *APLNR*, and *EDN1*) and HAPE susceptibility (Table 1), while the exact functions and mechanisms of most of the above-mentioned genes in HAPE pathogenesis need to be further addressed.

##### Sympathetic overactivity

During exposure to acute hypoxia, HAPE-prone individuals exhibited exaggerated sympathetic activation, facilitating pulmonary vasoconstriction and alveolar fluid flooding (Fig. 3, left).^218^ Importantly, sympathetic overactivation was directly associated with exaggerated hypoxic pulmonary hypertension and preceded the development of pulmonary edema.^218^ These findings suggest that hypoxia-induced sympathetic overactivity may contribute to the development of exaggerated pulmonary hypertension in HAPE-prone subjects. Consistent with this concept, during high-altitude exposure, α-adrenergic blockade improved hemodynamics and oxygenation by effectively reducing PAP compared with non-specific vasodilators or oxygen.^219^ Targeting the sympathetic nervous system may be a promising strategy for preventing HAPE. Evidence revealed that NO primarily reduced basal sympathetic vasoconstrictor tone rather than excitability, indicating that defective NO synthesis may lead to sustained vasoconstriction, which is mediated by exaggerated sympathetic activation, causing pulmonary hypertension in subjects susceptible to HAPE.^220^

##### Defective NO synthesis

Both endothelial NO synthase (eNOS) and inducible NO synthase (iNOS) are responsible for converting L-arginine to L-citrulline and NO in an oxygen-dependent manner.^221^ Upon hypoxia challenge, activated HIF-1/2 enhances expression of iNOS and eNOS, resulting in increased NO production.^222^ Actually, the NO production in the lung paradoxically decreases, potentially attributed to reduced availability of oxygen required for NO synthesis. NO is a vasodilator crucial for regulating pulmonary vascular tone.^223^ In addition to its direct effects, NO also reduces oxidative stress, which exacerbates HPV, thereby alleviating hypoxic pulmonary hypertension.^224^ Therefore, in NO-deficient states, both attenuated vasodilation and augmented oxidative stress contribute to exaggerated hypoxic pulmonary hypertension.

Previous research revealed that exhaled NO by high-altitude adapted populations, such as Tibetans residing at 4200 m and Bolivian Aymara at 3900 m, was significantly higher than in the low-altitude American populations.^225^ Additionally, compared to low-altitude Americans, the circulating concentrations of bioactive NO products were greater in Tibetan highlanders.^226^ Elevated NO levels in high-altitude populations suggested that increasing NO concentration might serve as a strategy to offset physiological hypoxia by facilitating pulmonary vasodilation.^227^ Furthermore, in HAPE-susceptible individuals exposed to short-term hypoxia, exhaled NO was significantly lower than resistant, and the exhaled NO was inversely related to PAP (Fig. 3, middle).^228,229^ These findings suggest that defective pulmonary epithelial NO synthesis may contribute to exaggerated hypoxic pulmonary hypertension and subsequent pulmonary edema in HAPE-prone subjects. Importantly, genetic studies supported that HAPE susceptibility was associated with eNOS polymorphisms and impaired vascular NO synthesis in certain populations.^120,121^

At high altitude, NO inhalation dramatically reduced sPAP and significantly improved arterial oxygenation, accompanied by redistribution of blood flow from edematous to nonedematous areas in HAPE-susceptible individuals compared with resistant subjects.^230^ Increasing NO availability by administration of the phosphodiesterase-5 (PDE5) inhibitor tadalafil decreased sPAP to prevent pulmonary edema in a small cohort of HAPE-prone people.^231^ Tadalfil also reduced HAPE incidence in adults with prior HAPE. These findings further support the pivotal involvement of impaired pulmonary endothelial NO synthesis in HAPE pathogenesis.

##### Augmented ET-1 synthesis

ET-1, a potent vasoconstrictor, is also synthesized by the pulmonary endothelium. Studies showed that exposure to high altitudes increased plasma ET-1 concentration in healthy volunteers.^163,232^ Individuals prone to HAPE exhibited higher plasma ET-1 levels compared to resistant individuals (Fig. 3, middle).^233^ Moreover, as ET-1 levels increased during high-altitude exposure, AMS severity worsened, suggesting ET-1 as a potential independent predictor of AMS occurrence and severity.^163^ These findings indicate that excessive ET-1 is an important factor in HAPE pathogenesis. A study involving 34 mountaineers revealed that marked increase in plasma ET-1 level was associated with elevated sPAP after ascent (Fig. 3, middle).^234^ Another research also delineated that PAP was positively correlated with increased plasma ET-1, indicating the contribution of elevated ET-1 to exaggerated pulmonary hypertension at high altitude.^233^ However, how ET-1 affects HAPE development remains to be further investigated. Bosentan, an ET-1 antagonist, obviously blunted the increase of sPAP induced by acute hypoxic exposure.^235,236^ However, its use requires caution due to decreased urinary volume and free water clearance.^235^ These results indicate that the prophylactic benefits of ET-1 antagonism against altitude-induced pulmonary hypertension may be accompanied by impaired volume adaptation.

Interestingly, during hypoxia exposure, crosstalk between ET-1 and NO exerts opposing effects on vascular tone. NO, an endothelium-derived relaxing factor, suppressed hypoxia-induced ET-1 expression and synthesis.^237^ This implies that hypoxia disrupts the ET-1/NO balance, leading to defective NO synthesis and augmented ET-1 production, which are causally linked to exaggerated pulmonary hypertension and HAPE pathogenesis. Indeed, ET-1 binds endothelin A (ETA) receptors, causing vasoconstriction, and endothelin B (ETB) receptors, causing vasodilation (via triggering NO release).^238^ However, the exact effects and mechanisms of hypoxia on modulating ET-1 binding bias remain unclear. These findings suggest an intricate interplay between ET-1 and NO in pulmonary vascular regulation during high-altitude exposure, which is relevant for developing HAPE prevention/treatment strategies.

#### Inflammatory response

Under acute hypobaric hypoxia, ROS levels were significantly upregulated in HAPE patients or rats, indicating the possible role of oxidative stress in hypoxia-induced transvascular leakage.^239–241^ Clinical cohorts also revealed the involvement of oxidative stress pathway-related genes (*CYBA*, *GSTP1*, *EPAS1*, and *EGLN1*) polymorphisms in elevated HPAE risk (Table 1). Elevated free radicals, one form of ROS, were directly correlated with systemic rise of 3-nitrotyrosine (3-NT) and sPAP in HAPE individuals.^240^ These findings emphasize the crucial role of ROS and subsequent inflammatory responses in HAPE pathology. Intermedin (IMD)/adrenomedullin-2 was dramatically upregulated in murine lung and pulmonary microvascular endothelial cells (PMECs) after acute hypoxia (HIF-1α-dependent).^242^ Hypoxia-mediated elevation of IMD stabilized endothelial barrier function by suppressing permeability in human lung microvascular endothelial cells (HMVEC-Ls) and isolated lungs, suggesting its potential for HAPE intervention.

There is a long-standing debate concerning the involvement of inflammation in alveolar capillary leakage in HAPE. Several studies, conducted in individuals and animal models with established HAPE, showed raised concentrations of proinflammatory mediators (such as NF-κB), cytokines (such as TNF-α and IL-6), and chemokines (such as MIP-1 and MCP-1) in blood or bronchoalveolar lavage fluid (Fig. 3, right).^241,243,244^ These observations strongly suggest that inflammation, especially mediated by proinflammatory cytokines and chemokines, may be involved or even a causal factor in HAPE pathogenesis via aggravating the permeability of the lung microvasculature. However, Swenson et al. argued that inflammation might not be a primary event or causal factor in HAPE pathogenesis, since there were no significant differences in bronchoalveolar lavage levels of neutrophils, proinflammatory cytokines (IL-1β, IL-8 and TNF-α), and eicosanoids between subjects resistant and susceptible to HAPE.^245^ The discrepancies of inflammation as an initiator in HAPE development can be attributed to the timing of lavage. In earlier studies, bronchoalveolar lavage was conducted after HAPE was well-established, typically 1–2 days post-onset. In contrast, Swenson and colleagues performed bronchoscopy very early in the course of the illness, often within 3–5 h. Hence, Swenson and colleagues postulated that inflammation is possibly a secondary response to alveolar–capillary barrier disruption or edema. This argument was supported by prospective studies measuring inflammatory markers, which showed no signs of inflammation before or at the onset of HAPE.^246,247^ Ultimately, the excessive inflammation induced by hypoxia can exacerbate pulmonary capillary leakage by causing lung endothelial damage.^244^

In contrast, there is evidence that challenges this typical argument that inflammatory reactions exhibit as a secondary response in HAPE progression.^248^ Exposing the rats to hypobaric hypoxia at 9142 m for 5 h, the proinflammatory molecules (such as TNF-α and IL-6) and mediators (such as NF-κB) remarkably increased. These findings indicate that inflammation may be an initiating event in HAPE-prone subjects. Furthermore, not all cases of HAPE presented evidence of inflammation in alveolar lavage fluid, implying that inflammatory response is not essentially associated with HAPE susceptibility or development.^243,249^ Therefore, whether and how inflammation contributes to HAPE pathogenesis are still matters of debate, which need additional investigations with a larger number of subjects to comprehensively address this topic. In addition, it should be noted that what mechanisms underlie activation of secondary inflammation as well as inflammation-mediated HAPE pathogenesis still warrant further investigation.

Although the inflammatory basis of HAPE pathophysiology is not yet clear, the implementation of anti-inflammatory approaches successfully ameliorated HAPE in susceptible individuals/animals, suggesting that the significant increase in proinflammatory cytokines and chemokines assumes a pivotal role in pathogenic processes of HAPE.^248,250,251^ Besides, people who were constitutionally resistant to HAPE may develop this disorder because of enhanced pulmonary capillary permeability mediated by virus-induced inflammation, which mechanism was supported by the evidence from animal studies with endotoxins or viruses challenge.^252–254^ Hence, inflammation possibly acts as a secondary response to alveolar edema and microvascular disruption, and subsequently contributes to HAPE susceptibility and development via triggering greater pulmonary capillary permeability.

#### Reduced alveolar fluid clearance

Although the initial disruption of the alveolar‒capillary barrier and subsequent fluid leakage are recognized as the primary triggers, caused by exaggerated hypoxic pulmonary hypertension and excessive inflammation, impaired alveolar fluid clearance is increasingly recognized as a critical factor in HAPE development.^255^ Active sodium (Na^+^) transport across the alveolar epithelium is crucial for preventing fluid accumulation, mediated by epithelial Na^+^ channels (ENaC) and Na^+^-potassium (K^+^) pump (Na^+^-K^+^-ATPase).^256^ Subsequently, active Na^+^ transport generates an osmotic gradient to drive water out of the alveolar spaces through aquaporin (AQP) 1 and 5 (AQP1 and AQP5).^257^ Since direct measurement of alveolar Na^+^ transport activity is not feasible, the nasal potential difference is usually adopted to represent alveolar Na^+^ transport activity. HAPE-susceptible individuals exhibited lower nasal transepithelial potential differences than resistant, implying impaired transepithelial Na^+^ transport in HAPE-prone subjects (Fig. 3, right).^258,259^

Indeed, under hypoxic condition, both expression and activity of ENaC and Na^+^-K^+^-ATPase were downregulated in affected animals, thus dramatically diminishing transepithelial Na^+^ transport to further suppress alveolar liquid clearance (Fig. 3, right).^260,261^ Impaired β2-adrenergic receptor signaling pathway may be responsible for the reduction in the activity of ENaC and Na^+^-K^+^-ATPase during hypoxia challenge.^262^ Salmeterol, a long-acting β2-adrenergic agonist, was used to identify whether pharmacological enhancement of transepithelial Na^+^ transport can reduce HAPE incidence in susceptible individuals.^258^ Prophylactic salmeterol inhalation upregulated ENaC and Na^+^-K^+^-ATPase, thereby improving alveolar fluid clearance and decreasing HAPE incidence by more than 50% compared to the placebo group. However, the hemodynamic effects of salmeterol may also prevent HAPE, so its specific contribution to improved alveolar fluid clearance remains uncertain.^263,264^ In vivo, AQP5 deficiency or partial ENaC deficiency depressed alveolar fluid clearance, exacerbating hypoxia-induced pulmonary edema and lung injury.^265,266^ In conclusion, these findings support that alveolar fluid clearance is a critical defense mechanism against HAPE development.

Additionally, multiple cohorts suggest potential contributions of polymorphisms in alveolar stability/function-related genes (*ADRB2*, *TIMP3*, *SFTPA1*, and *SFTPA2*) to HAPE pathogenesis (Table 1). However, their exact functions and mechanisms remain unclear.

### Pathogenic mechanisms of CMS

**Fig. 4:**
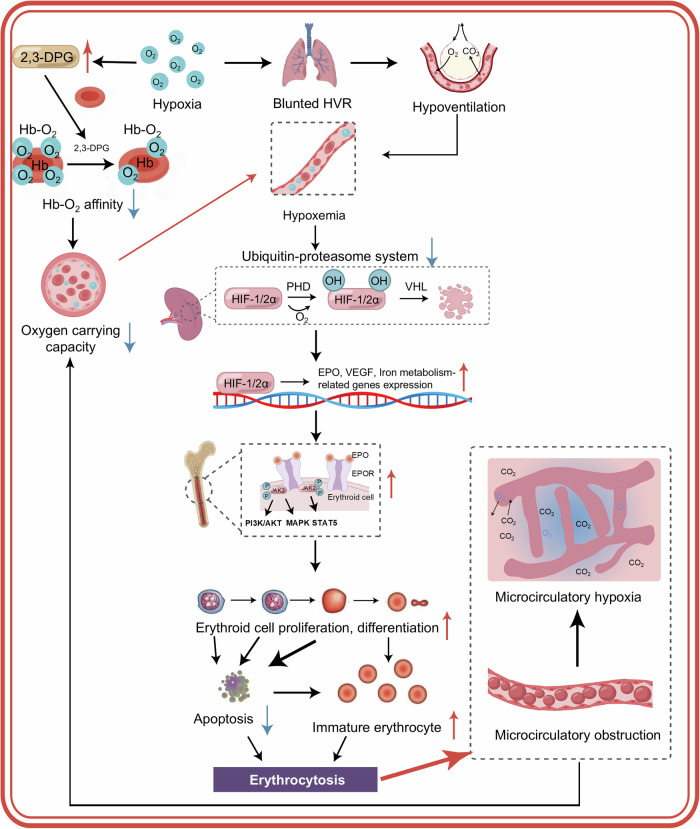
Pathophysiology of CMS. Hypoventilation (due to blunted HVR) and elevated 2,3-DPG (reducing Hb-O₂ affinity and oxygen-carrying capacity) collectively contribute to the development and worsening of hypoxemia. Hypoxemia stabilizes HIF-1/2α by suppressing its hydroxylation, thereby upregulating the expression of EPO, VEGF, and iron metabolism-related genes. EPO then binds to its receptor (EPOR) on erythroid progenitors, activating signaling pathways (PI3K/AKT, MAPK, and STAT5) to inhibit apoptosis and promote proliferation/differentiation. This results in the accumulation of RBCs, microcirculatory obstruction, localized tissue hypoxia, and further aggravation of systemic hypoxemia, thereby perpetuating a pathological feedback loop

The underlying mechanism of CMS pathogenesis is still largely elusive. Chronic hypoxia has been identified as the fundamental cause of CMS via initiating a complex series of physiological and pathological responses. Mounting evidence has gradually revealed the precise mechanisms underlying the occurrence and development of CMS (Fig. 4). In brief, the primary pathogenic mechanism in CMS is respiratory in origin. CMS development is driven primarily by hypoventilation due to a blunted hypoxic ventilatory response (HVR), leading to hypoxemia and an increase in RBC mass. Additionally, severe hypoxemia triggers an excessive production of 2,3-diphosphoglycerate (2,3-DPG), causing a rightward shift of the oxygen dissociation curve. This reduces Hb-O_2_ affinity, thereby lowering SaO₂ and ultimately diminishing overall oxygen-carrying capacity, further exacerbating hypoxemia. Hypoxemia, driven by hypoventilation and excessive 2,3-DPG, promotes EPO expression by inhibiting HIF-1/2α hydroxylation and subsequent degradation. Circulating EPO availability rather than its concentration then binds to the EPO receptor (EPOR) on erythroid progenitors to suppress their apoptosis while enhance their survival, proliferation, and differentiation into erythrocytes, leading to erythroid expansion. This excessive erythrocytosis is a compensatory mechanism to increase the oxygen-carrying capacity of the blood in high-altitude environments, which in turn could exacerbate hypoxemia when the erythropoietin response becomes excessive by impairing microcirculation. It is worth mentioning that the current understanding of CMS pathogenesis is based primarily on correlational studies, which are often conducted with limited sample sizes. Moreover, the role of blunted HVR or hypoventilation in CMS development warrants further investigation, particularly given the significant individual variability observed.

The hypoventilation caused by blunted HVR is considered a primary mechanism underlying exacerbated hypoxemia and subsequent excessive erythrocytosis, consequently leading to CMS development (Fig. 4, middle).^267^ Pioneering studies showed that the partial pressure of arterial CO_2_ (PaCO_2_) slightly increased in CMS subjects. Lozano et al. reported higher PaCO_2_ in 6 high-altitude residents with excessive erythrocytosis (34.8 mmHg) than 6 healthy individuals (31.9 mmHg) at the same altitude.^268^ Meanwhile, Cruz et al. also reported elevated PaCO_2_ in 22 CMS patients (38.1 mmHg) compared with controls (32.5 mmHg).^269^ These findings suggest the hypoventilation may contribute to CMS pathogenesis. Furthermore, several studies demonstrated that the probable cause of hypoventilation was related to blunted HVR. Researchers delineated the higher central and peripheral chemoreflex set points in susceptible individuals, leading to lower ventilatory sensitivities to PaCO_2_ in CMS patients than non-CMS controls (Fig. 4, middle).^270,271^ The blunted HVR and resultant hypoventilation were remarkably correlated with exacerbated hypoxemia (decreased SpO_2_) and excessive erythrocytosis (increased Hct). However, blunted HVR does not fully explain excessive erythrocytosis, as significant variability exists: not all CMS patients exhibit hypoventilation or severe hypoxemia, and even healthy non-CMS individuals may experience hypoventilation.^271^ These results highlight the complexity of factors contributing to CMS pathogenesis.

The reduced Hb-O_2_ affinity, which decreases the oxygen-carrying capacity, plays a pivotal role in exacerbating hypoxemia and excessive erythrocytosis. This affinity is negatively related to the concentration of 2,3-DPG and positively associated with pH level. Ge et al. have demonstrated that compared to non-CMS individuals at 4300 m, patients with CMS (13 cases) exhibited significantly elevated levels of 2,3-DPG and PaCO_2_, concurrent with decreased pH and PaO_2_ (Fig. 4, left).^59^ Elevated 2,3-DPG levels were inversely correlated with PaO_2_. It should be noted that an appropriate increase in 2,3-DPG level could facilitate the release of oxygen to tissues by reducing Hb-O_2_ affinity, whereas excessive levels decrease the overall oxygen-carrying capacity and exacerbate hypoxemia. A recent study also indicated that the level of 2,3-DPG in the bone marrow supernatant and serum was significantly higher in the CMS group (20 patients) compared to the controls, suggesting the elevated level of 2,3-DPG might play a crucial role in CMS pathogenesis (Fig. 4, left).^272^

Hypoxemia, resulting from blunted HVR-induced hypoventilation and elevated 2,3-DPG, inhibits the hydroxylation of HIF-1/2α to enhance its stabilization and accumulation.^273^ Ge’s group reported that the expression levels of both VHL and HIF-2α were higher in CMS patients than controls, indicating the involvement of the VHL-HIF-2α axis in hypoxemia-triggered CMS pathogenesis.^274^ Moreover, HIF-1/2α bind to promoters to enhance EPO, VEGF, and iron metabolism-related genes expression, which appears to contribute to CMS development.^275,276^ Circulating EPO then binds to EPOR on the surface of erythroid progenitors in the marrow to trigger the activation of the PI3K/AKT, MAPK and STAT5 signaling pathways, resulting in the protection of cells from apoptosis as well as increased survival, proliferation and ultimate differentiation into erythrocytes.^277,278^ However, several studies have shown that excessive erythrocytosis is not strictly dependent on severe hypoxemia or EPO level at high altitudes. Despite the increased relative risk of excessive erythrocytosis with severe hypoxemia at 4340 m in Cerro de Pasco, Peru, a significant proportion (27%, n = 965) of highlanders with normal SpO_2_ levels exhibited excessive erythrocytosis, whereas some (28%) with low SpO_2_ levels maintained normal Hb levels.^64^ Additionally, another study reported that 47% of subjects without excessive erythrocytosis had high serum EPO.^279^ These findings underscore the individual variability in high-altitude adaptation and suggest a complex interplay between EPO signaling, hypoxia sensitivity, and erythroid progenitor response. Subsequent reports have shifted the focus from EPO concentration to EPO availability, measured by the ratio of serum EPO to its soluble receptor sEPOR, an endogenous antagonist of EPO action, in excessive erythrocytosis development (Fig. 4, middle).^57,279^ They found that a lower sEPOR level and higher EPO-to-sEPOR ratio correlated well with Hb level, indicating a more potent erythropoietic stimulus even at similar EPO concentrations. Besides, these findings provide an explanation for the previously observed lack of correlation between EPO concentration and excessive erythrocytosis in highlanders.

Furthermore, recent research has indicated that erythroid progenitors from individuals with excessive erythrocytosis exhibit an enhanced proliferative response under hypoxic conditions, along with an upregulation of genes involved in erythropoiesis.^280^ These findings suggest that the erythroid progenitors of CMS patients may be genetically predisposed to excessive erythrocytosis development. In addition, human studies demonstrated that the excessive Sphingosine-1-phosphate (S1P) and oxidative stress markers (e.g., ascorbate radicals [A•-], 8-iso-PGF2α, and malondialdehyde (MDA)) were more pronounced in CMS patients/rats.^281–285^ However, the precise mechanisms need to be further explored.

Unexpectedly, genetic variations in several HIF pathway-related genes (*EGLN2*, *EGLN3*, *HIF1A*, and *VHL*, except *EGLN1*) and even EPO pathway-associated genes (EPO and EPOR) were not associated with CMS susceptibility, indicating the need to elucidate the exact mechanisms in CMS pathogenesis (Table 1). Besides, the precise function and mechanism of vascular homeostasis-related genes (*ACE*, *AGT*, and *VEGFA*) polymorphisms require additional investigations (Table 1).

### Pathogenic mechanisms of HAPH

**Fig. 5:**
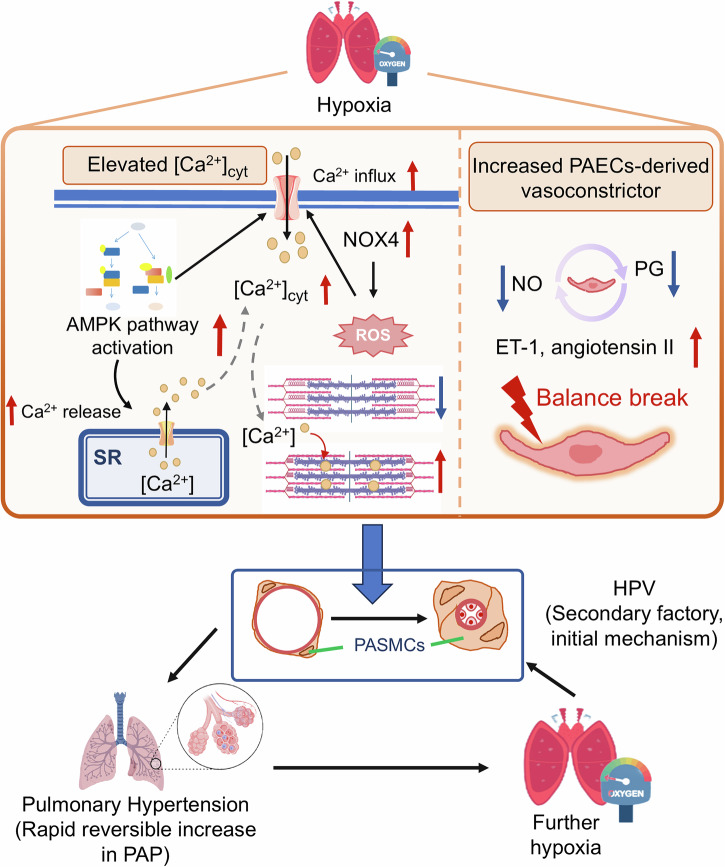
PASMCs constraction-mediated HPV is an initial mechanism of HAPH pathogenesis. On the one hand, elevated cytoplasmic Ca²⁺ concentration in PASMCs is driven by enhanced calcium influx (mediated by increased ROS production, Kv channel inhibition, and AMPK activation) and SR Ca²⁺ release (mediated by upregulated SR Ca²⁺ channels and AMPK activation). On the other hand, hypoxia induces PAECs dysfunction, characterized by increased production of the vasoconstrictor ET-1 and decreased synthesis of the vasodilator NO. These mechanisms synergistically enhance PASMCs contraction, thereby triggering HPV. As a secondary factor for PAP elevation in HAPH, HPV induces rapid and reversible pulmonary vasoconstriction and elevated PAP, leading to pulmonary hypertension, while further hypoxia perpetuates this pathological cycle

**Fig. 6:**
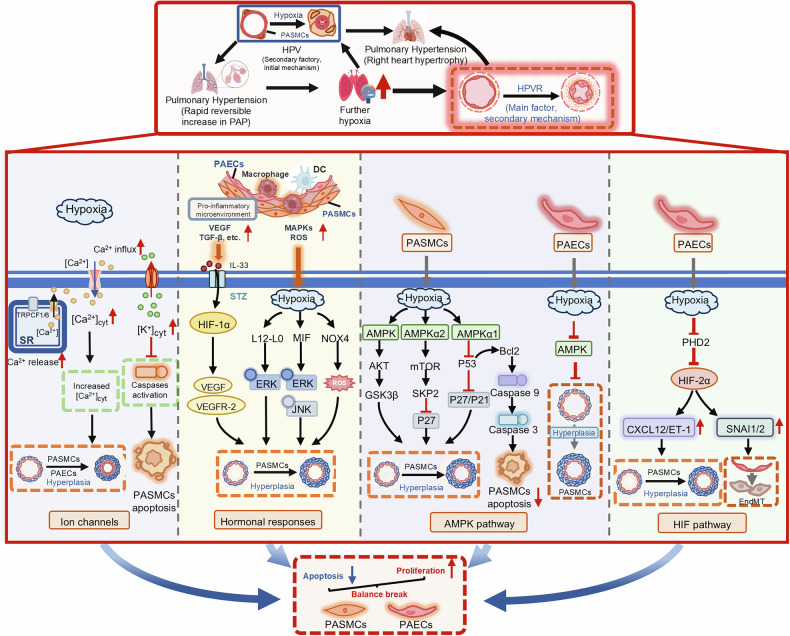
Pulmonary vascular remodeling is a secondary mechanism of HAPH pathogenesis. Furthermore, hypoxia drives HPVR, the main factor in HAPH progression. Multiple mechanisms act concurrently: hypoxia modulates ion channel activity or expression, increasing cytoplasmic [Ca²⁺] and [K⁺] ion channel activity in PASMCs and PAECs to promote their proliferation and inhibit PASMCs apoptosis; hormonal/inflammatory responses (e.g., VEGF, TGF-β, ROS, and MAPKs) enhance PASMCs proliferation by promoting VEGF/VEGFR2 expression and activating the ERK and JNK pathways; hypoxia-enhanced activation of the AMPK-Akt-GSK3β, AMPKα1-P53-P27/P21, and AMPKα2-mTOR-Skp2-P27 axes mediate PASMCs proliferation, while the AMPKα1-P53-Bax/Bcl-2-caspase-9-caspase-3 pathway suppresses PASMCs apoptosis, and AMPK activation in PAECs also fosters PASMCs proliferation; and the activation of the PHD2-HIF-2α pathway in PAECs upregulates CXCL12/ET-1 and SNAI1/2 expression, promoting PASMCs proliferation and EndMT, respectively. Collectively, these mechanisms synergistically disrupt the balance between proliferation-apoptosis in PASMCs and PAECs, facilitating the remodeling of small and micro pulmonary arteries. This further aggravates hypoxia-induced pulmonary hypertension progression, ultimately leading to right heart hypertrophy

Chronic hypoxia is the initiating factor of HAPH pathogenesis, but the underlying mechanisms remain poorly understood. Growing evidence has progressively unveiled the mechanisms involved in HAPH development (Figs. 5 and 6). In short, HAPH is fundamentally characterized by vascular disturbances. The initial mechanism of HAPH pathogenesis is HPV (Fig. 5), which decreases the vascular lumen diameter and increases pulmonary vascular resistance, resulting in redistribution of more blood flow to better-oxygenated areas to optimize ventilation‒perfusion matching. HPV triggers widespread pulmonary arterial constriction, leading to a rapid and reversible increase in PAP and culminating in pulmonary hypertension. Hypoxia-induced increases in intracellular Ca^2+^ concentration and subsequent endothelium-dependent modulation contribute to promoting pulmonary arterial SMCs (PASMCs) contraction and resultant HPV. However, HPV is now considered a secondary factor in elevated PAP since oxygen administration reduces PAP by only 15% to 20%.^286^ Previous reports have indicated structural remodeling of the distal pulmonary arteries and arterioles, which are normally devoid of SMCs. Notably, the thickening of the media layer of the pulmonary arterioles and the muscularization of normally non-muscularised small arteries persist even after returning to normoxia, along with perpetuation of elevated PAP. These findings indicate that hypoxic pulmonary vascular remodeling (HPVR) in originally weakly muscularized arterioles and normally non-muscular pulmonary vessels is likely the main factor responsible for HAPH development (Fig. 6). Conclusions on PASMCs/pulmonary arterial endothelial cells (PAECs) hyperproliferation are derived from in vitro studies, while muscular thickening data rely on in vivo rodent models. The ion channels, hormonal responses (transforming growth factor-beta (TGF-β), VEGF, and oxidative biomarkers), along with adenosine monophosphate-activated protein kinase (AMPK) and HIF pathways, are involved in HPVR via modulating the proliferation and/or apoptosis of PASMCs and PAECs.

#### PASMCs contraction-mediated HPV

##### Elevated Ca^2+^ concentration

Hypoxia-induced augmented PASMCs contraction and HPV are mediated primarily by an increase in intracellular Ca^2+^ concentration. The increased Ca^2+^ concentration in PASMCs was attributed mainly to chronic hypoxia-induced inhibition of voltage-gated potassium (K^+^) (Kv) channels activity and expression, which lead to membrane depolarization and extracellular Ca^2+^ entry mainly through voltage-dependent Ca^2+^ channels (VDCC), consequently resulting in PASMCs contraction and pulmonary vasoconstriction (Fig. 5, left).^287–289^ Interestingly, the hypoxia-mediated suppression on Kv activity and expression was unique to pulmonary vasculature not systemic arteries, thus specifically triggering pulmonary vasoconstriction.^289,290^ In addition, another source of elevated Ca^2+^ levels in PASMCs was Ca^2+^ release from the intracellular sarcoplasmic reticulum, which was associated with the development and maintenance of pulmonary vasoconstriction (Fig. 5, left).^291,292^ This secondary increase in Ca^2+^ is mediated by store-operated calcium channels (SOCCs) and receptor-operated calcium channels (ROCCs), consisting of acid-sensing ion channels (ASICs), Orai, stromal interaction molecule (Stim), and transient receptor potential channels (TRPCs).^293^ Under chronic exposure to hypoxia, the expression of Orai1 and 2, TRPC1 and 6, Stim1 and 2, and ASIC1 were upregulated, which might be modulated by H_2_O_2_ production and HIF-1α, causing the increased Ca^2+^ concentration.^293–296^ Furthermore, the Orai1 channels, TRPC6 and Stim1 exhibited a critical role in pulmonary hypertension, highlighting the crucial role of Ca^2+^ release from the sarcoplasmic reticulum in enhanced PASMCs contraction and subsequent pulmonary vasoconstriction.^296,297^

##### Endothelium-derived vasoactive mediators

Although PAECs are not directly involved in the vasoconstriction associated with chronic hypoxia-induced pulmonary arterial hypertension, they exert a pivotal role in modulating PASMCs contraction by secreting redundant endothelium-derived mediators, encompassing vasodilators (e.g., NO and prostacyclin (PGI_2_)) and vasoconstrictors (e.g., ET-1).^238^ Under chronic hypoxia, the elevated ET-1 concentration was mediated by HIF-1α-induced TRPC1 and 6, Orai2 and Stim2 (Fig. 5, right).^293,294,297^ ET-1 exhibits a bifunctional role in HAPH progression: a vasoconstrictor via binding to ETA and a vasodilator through binding to ETB causing NO release. Notably, studies revealed that the vasoconstrictive and vascular-remodeling impacts of ET-1 manifested within PASMCs (Fig. 5, right).^70^ This was supported by both in-vivo and in-vitro findings that macitentan (a dual ETA/ETB antagonist) or BQ788 (a ETB antagonist) remarkably ameliorated pulmonary arterial hypertension by modulating the vasomotor tone, PASMCs metabolism and proliferation, as well as pulmonary and systemic perfusion and angiogenesis.^298^ Besides, during chronic hypoxia, decreased NO bioavailability, mediated by hypoxia-induced significant increase in superoxide radicals, also seemed to be related to augmented pulmonary vasoconstriction to exaggerate pulmonary hypertension (Fig. 5, right).^299,300^ Nevertheless, these two studies reported conflicting findings regarding the eNOS expression in PAECs under chronic hypoxia, warranting further investigations. Additionally, the contribution and mechanism of another vascular homeostasis-related gene, ACE, in HAPH pathogenesis need to be further addressed since its polymorphism was associated with HAPH susceptibility (Table 1).

##### Oxidative responses

During chronic hypobaric hypoxia, the elevated production of ROS and other oxidative biomarkers were elevated in HAPH patients or rats, providing potential biomarkers for the prevention and treatment.^281–284,300–304^ During hypoxia, ROS generation was elevated for diminished tricarboxylic acid (TCA) cycle, leading to HIF-1α-mediated inhibition of Kv channel, membrane depolarization, Ca²⁺ influx, and subsequent enhanced PASMC contraction and HPV (Fig. 5, left).^305^ In addition, there is evidence that directly support the roles of ROS in HAPH. NOX4 may be one of the major sources of ROS produced in the pulmonary artery, such as O_2_^−^ or H_2_O_2_.^306^ It has been reported that Nox4 was significantly upregulated in HAPH (Fig. 5, left).^299–301,307^ Further studies have confirmed the direct contribution of NOX4 to HAPH pathogenesis. Suppressing NOX4 using siRNA or pharmacological inhibitors could reduce ROS generation. This reduction enhanced PASMCs remodeling and contraction through promoting proliferation, migration, and elastin deposition, as well as by increasing STIM1-mediated Ca^2+^ influx, respectively, thereby contributing to HAPH development (Fig. 5, left; Fig. 6, middle, hormonal responses).^307–310^

##### AMPK pathways

AMPK plays a major role in regulating the energy balance in eukaryotic cells and is activated during nutrient starvation, especially hypoxia.^311^ AMPK is composed of a catalytic subunit (α subunit (α1/α2)) and two regulatory subunits (β (β1/β2) and γ (γ1/γ2/γ3)).^312^ Studies by Evans et al. revealed strong correlations between HPV and hypoxia-mediated AMPK activation, which can elicit cyclic ADP‒ribose (cADPR)-dependent Ca^2+^ release from the sarcoplasmic reticulum in PASMCs (Fig. 5, left).^313,314^ Besides, Evans et al. elucidated the causal role of AMPK in HPV pathogenesis by using a nonselective AMPK antagonist or conditional deletion of AMPK α1/α2 with CRISPR gene editing.^315,316^ Furthermore, HPV was suppressed through targeted deletion of either AMPK α1 or AMPK α2 in pulmonary arterial myocytes, whereas the hypoxia-mediated reduction of Kv channel activity was blocked by AMPK α1 deletion alone (Fig. 5, left).^316^ These results suggest that hypoxia-induced AMPK α1 activation can increase intracellular Ca^2+^ concentration by inhibiting Kv channel activity to augment PASMCs contraction and pulmonary vasoconstriction, contributing to initiation and progression of HAPH. However, the exact mechanisms by which AMPK α2 supports HPV are not yet fully understood, requiring additional investigations for clarification. Additionally, Evans et al. revealed that AMPK activation, triggered by hypoxia-inhibited mitochondrial oxidative phosphorylation, induced Ca^2+^ influx into carotid body glomus cells to enhance sensory afferent discharge, thus further exacerbating HPV.^313,314^

#### Pulmonary vascular remodeling

##### Ion channels

Emerging evidence has shown that upon hypoxia challenge, the altered activity of ion channels also plays key roles in HPVR and resultant pulmonary hypertension. Membrane depolarization, caused by hypoxia-induced downregulation of Kv channel activity and expression, triggered Ca^2+^ influx and release, which was also involved in PASMCs proliferation via modulating transcription of proliferative genes (Fig. 6, left, ion channels).^317,318^ Synergistically, high levels of intracellular K^+^, mediated by hypoxia-induced suppression of Ca^2+^-sensitive voltage-dependent K^+^ (K_Ca_) and Kv channels activity in PASMCs, remarkably attenuated cytoplasmic caspases activation to inhibit apoptosis and promote cell survival (Fig. 6, left, ion channels).^287,319^ Collectively, under hypoxic conditions, the elevated intracellular Ca^2+^ concentration and reduced K^+^ efflux exhibit proliferative and antiapoptotic effects, respectively, thereby contributing to the HPVR and pulmonary hypertension. Furthermore, except Kv channel, enhanced SOCCs activation, caused by hypoxia-mediated upregulation of TRPC channels such as TRPC1 and TRPC6, were also involved in increasing intracellular Ca^2+^ level in PASMCs (Fig. 6, left, ion channels).^320–322^ As mentioned above, elevated Ca^2+^ concentration also contribute to hypertrophy and hyperplasia of PASMCs, consequently aggravating hypoxia-induced pulmonary hypertension. Additionally, chronic hypoxia dramatically upregulated TRPC4 expression to augment SOCCs-mediated increase of Ca^2+^ concentration to promote PAECs proliferation, subsequently exacerbating HPVR and pulmonary hypertension (Fig. 6, left, ion channels).^323,324^ Notably, what mechanisms underlie altered ion channel activity-mediated HPVR still warrant further investigation.

##### Hormonal responses: inflammatory cytokines

Extensive research has demonstrated that hormonal responses also play pivotal roles in the formation and development of HAPH. In HAPH, inflammatory pathways may contribute to HPVR progression by modulating the proliferation of PASMCs and PAECs, as well as facilitating Endothelial-to-Mesenchymal Transition (EndMT).^325^ Upon chronic hypoxic exposure, there was a robust and persistent accumulation of inflammatory cells, such as monocytes and dendritic cells, within and around the vessel wall, following the upregulation of hypoxia-induced inflammatory factors, such as IL-1β, IL-33, CXCL12/SDF-1, TGF-β, and IL-6, as well as VEGF (Fig. 6, middle, hormonal responses).^326^ VEGF was particularly implicated in inducing SDF-1,^327^ indicating its role in the chronic hypoxia-induced proinflammatory microenvironment associated with HPVR. Previous reports also showed obvious increase in expression of VEGF and its receptor VEGF receptor 2 (VEGFR-2/ Flk/KDR) in patients and animal models with hypoxic pulmonary hypertension,^328,329^ which was HIF-1α dependent (Fig. 6, middle, hormonal responses).^330^ Inhibition of VEGFR-2-mediated signaling could obviously alleviate HPVR and pulmonary hypertension by suppressing PAECs proliferation, underscoring the contribution of abundantly expressed VEGF and VEGFR-2 in the lung under hypoxic conditions to HPVR progression and HAPH pathogenesis.^329^ Besides, the expression of IL-33 and its receptor ST2 was significantly elevated in PAECs from humans and mice subjected to chronic hypoxia, which in turn partly aggravated HPVR by triggering upregulated production of HIF-1α, VEGF and VEGFR-2, leading to HAPH pathogenesis (Fig. 6, middle, hormonal responses).^331^ The expression of TGF-β, involved in increased EndMT of PAECs and subsequent pulmonary vascular remodeling, was remarkably elevated after hypoxia treatment.^332,333^ Importantly, upregulated TGF-β expression was observed only in animal and cellular models, with a lack of clinical data from patients with HAPH.

MAPKs, including the ERK1/2, JNK1/2/3, p38 (α, β, γ, and δ), and ERK5 branches, were also involved in inflammation,^334^ which in turn promoted PASMCs proliferation (Fig. 6, middle, hormonal responses). After sustained hypoxic exposure, the expression of 12-LO metabolite, 12(S)-hydroxyeicosatetraenoic acid [12(S)-HETE], was obviously elevated, resulting in enhanced proliferation of PASMCs of rats via inducing ERK1/2 phosphorylation but not p38 (Fig. 6, middle, hormonal responses).^335^ These findings indicate the potential contribution of 12-LO- ERK1/2 pathway to the development of HPVR and subsequent pulmonary hypertension. Besides, the expression of macrophage migration inhibitory factor (MIF), a critical proinflammatory mediator, was also upregulated in the lung tissues from pulmonary hypertensive rats subjected to chronic hypoxic exposure (Fig. 6, middle, hormonal responses).^336^ This study further revealed that MIF facilitated hypoxia-induced PASMCs proliferation through activating the ERK1/2 and JNK pathways, without the involvement of p38, thus contributing to HPVR and pulmonary hypertension.

##### Other mechanisms: the AMPK and HIF pathways

Currently, multiple studies have focused on the role of AMPK in pulmonary vascular remodeling by affecting the proliferation and apoptosis of PASMCs. One previous study revealed a significant increase of AMPKα1 and phosphorylated AMPKα1 in pulmonary arterioles, lung tissues (in vivo) and PASMCs (in vitro) during hypoxia.^337^ Administration with Compound C, an AMPK inhibitor, could markedly suppress the hypoxia-induced proliferation of PASMCs, indicating a detrimental role of AMPK in HAPH pathogenesis. Conversely, another study revealed that endothelial AMPK was obviously downregulated in patients and mice with hypoxia-induced pulmonary hypertension.^338^ Endothelial-specific AMPK knockout mice showed accelerated development of hypoxia-induced pulmonary hypertension by promoting PASMCs proliferation. However, these studies did not clarify the exact molecular mechanisms involved. A further report delineated distinct functions of α subunits of AMPK (α1 and α2) within PASMCs in hypoxia-induced pulmonary hypertension.^339^ Activation of AMPK α2 promoted PASMCs survival by maintaining myeloid cell leukemia-1 (MCL-1) expression, whereas AMPK α1 activation inhibited apoptosis through facilitating autophagy. These actions contributed to the vascular remodeling observed in hypoxia-induced pulmonary hypertension. Later studies revealed that the AMPK-Akt-GSK3β, AMPKα1-P53-P27/P21, and AMPKα2-mTOR-Skp2-P27 axes were involved in PASMCs proliferation, whereas the AMPKα1-P53-Bax/Bcl-2-caspase-9-caspase-3 contributed to PASMCs apoptosis (Fig. 6, middle, AMPK pathway).^340–342^ These findings underscore the complex roles and mechanisms of AMPK and its subunits in pulmonary vascular remodeling, providing potential therapeutic targets for HAPH.

Additionally, accumulating studies have indicated the pivotal role of HIF-mediated pathways in orchestrating the progression of HPVR. Heterozygous or partial deficiency of HIF1α or HIF2α (HIF1α^−/+^ or HIF2α^−/+^ mice) significantly protected against HAPH pathogenesis via delaying development of pulmonary vascular remodeling.^343–345^ Besides, partial HIF-2α deficiency was shown to abrogate the hypoxia-induced upregulation of ET-1 and plasma catecholamine levels, indicating that these HIF2α-mediated vasoconstrictors might be involved in HPVR progression.^345^ Subsequent reports further identified the specific contribution of endothelial HIF1α/HIF2α or myeloid HIF1α to HAPH pathogenesis. Endothelial HIF2α, but not HIF1α, was required for the development of pulmonary vascular remodeling and resultant pulmonary hypertension induced by chronic hypoxia.^346^ The HIF-2α-dependent elevation of arginase-1 might be responsible for this process through reducing NO production. Furthermore, some studies revealed that in PAECs, hypoxia-induced inactivation of prolyl-4 hydroxylase-2 (PHD2; encoded by the egl nine homolog (*EGLN1*) gene) enhanced PASMCs proliferation, partly through HIF-2α-mediated increase of CXCL12 and ET-1 expression, along with decrease in vasodilatory apelin receptor signaling, ultimately contributing to severe HAPH (Fig. 6, right, HIF pathway).^347,348^ Moreover, the endothelial PHD2-HIF-2α axis also played a crucial role in hypoxia-induced EndMT by modulating SNAI1/2 expression, causing vascular remodeling and severe HAPH (Fig. 6, right, HIF pathway).^349^ Notably, the deletion of endothelial HIF-1α or PASMCs-specific HIF-2α negligibly affected vessel muscularization and elevated PAP. Conversely, a recent study demonstrated that during chronic exposure to hypoxia, mice lacking myeloid-specific HIF-1α exhibited a significant decrease in right ventricular systolic pressure (RVSP), suggesting the contribution of myeloid-specific HIF-1α in the progression of pulmonary vascular remodeling and pulmonary hypertension.^350^ These studies delineated the distinct pathogenic roles and mechanisms of HIF1α and HIF2α in HAPH pathogenesis, providing novel therapeutic approaches for the treatment of this condition.

## Prevention and treatment strategies for altitude diseases

### Prevention of altitude illness

#### Nonpharmacological measures

##### Prevention of acute altitude illness: gradual ascent and preacclimatization

The prevention of high-altitude illness should take precedence over treatment. Despite limited controlled studies, slow and graded ascent is widely considered the most effective preventative measure, allowing sufficient time for acclimatization to changing altitudes.^351,352^ In particular, for AMS and HACE, sleeping altitude is more critical than daytime altitude; however, for HAPE, no prospective studies have confirmed that restricting sleeping altitude could prevent HAPE occurrence.^20^ Generally, at altitudes above 2500 m, it is commonly advised not to exceed an ascent rate of 500 m per day (based on sleeping altitude), with additional rest days scheduled for every 1000–1500 m of further ascent.^13,14,353^ Staging, another aspect of gradual ascent, involves staying at moderate altitudes of 2000–3000 m to facilitate acclimatization before subsequent rapid ascent.^354^ Both slow ascent and staging, which are traditional in mountaineering and trekking, reduce the risk of acute mountain illness and potentially improve exercise performance. However, time constraints and logistical challenges often preclude their use. Intermittent hypoxic exposure represents another potential preventive measure. The 2007 CATMAT recommendation advocates day trips to higher altitudes with overnight returns to lower altitudes for sleep, leveraging intermittent hypoxia for natural acclimatization.^355^

Given the time, logistical, and location limitations of traditional acclimatization, alternative strategies that mimic its effects have emerged. These simulated strategies include devices or chambers altering fractional inspired oxygen (FIO₂) or positive end-expiratory pressure (PEEP) to stimulate high-altitude conditions, offering attractive alternatives for climbers.^356–358^ The simulation of altitude hypoxia is frequently studied for intermittent hypoxia prevention. Research has yielded conflicting efficacy results, with some indicating benefits and others showing no clear effect, which could be attributed to the significant variations in hypoxic exposure protocols.^359–361^ Despite these discrepancies, longer and more frequent intermittent normobaric or hypobaric hypoxic exposures were generally suggested to reduce the risk of acute mountain illness better.^360–362^

In conclusion, although these preacclimatization strategies pose minimal risks and potential benefits, robust evidence supporting their efficacy is still lacking. More rigorous research is needed to establish optimal protocols and address implementation challenges, ensuring safety and effectiveness.

##### Prevention of chronic altitude illness: chronic intermittent exposure

Several studies proposed chronic intermittent high-altitude exposure as a preventive approach against excessive Hb concentration elevation.^363,364^ Long-term intermittent hypoxia induced a small but statistically significant increase in Hb values, which were higher than sea-level residents but lower than healthy high-altitude dwellers. Studies suggested that managing intermittent exposure could regulate Hb levels, preventing excessive elevations. Similarly, two recent studies have consistently revealed that intermittent short-duration reoxygenation may effectively prevent HAPH pathogenesis.^365,366^ They further indicated that the HIF-1α/NOX4/PPAR-γ axis was involved in regulating PASMCs proliferation induced by intermittent hypoxia. However, validation in human cohorts remains necessary.

#### Pharmacologic strategies

Several pharmacological agents are available for preventing acute high-altitude sickness (Table 2). However, pharmacological prophylaxis is not universally necessary; instead, the decision to initiate pharmacological prophylaxis should be based on an individual’s risk assessment.^11,351,352^ Specifically, for travelers at moderate-to-high-risk, including those with a history of HACE/HAPE or those ascending > 3000 m, prophylaxis should be strongly considered. Conversely, low-risk individuals (ascending <2500 m with no history of altitude illness) generally do not require prophylaxis. In contrast, pharmacologic prevention of chronic high-altitude illnesses (CMS and HAPH) is more challenging, with few effective preventive strategies available (Table 2), and pharmacological medication is still in the animal model stage. Below, we briefly describe the pharmacological agents and recommended dosages; Table 2 details their functions, contraindications, adverse effects, and indications.

**Table 2:** Medications for high altitude illness

Medication	Pharmacological function	Contraindications	Adverse events	Indications
Acetazolamide	As a sulfonamide, acetazolamide inhibits carbonic anhydrase, which reduces the reabsorption of bicarbonate in the kidneys.^464^ The increased bicarbonate excretion compensates for altitude-induced respiratory alkalosis and shifts toward metabolic acidosis, thereby optimizing respiratory drive and adaptation to high altitude. It also effectively improves altitude-related sleep disorders, primarily periodic breathing, further facilitating acclimatization to hypobaric hypoxia by enhancing sleep quality.^465^ Moreover, emerging research has revealed that acetazolamide can directly affect brain fluid balance by modifying aquaporin and Na channels to decrease water influx into cells.^466,467^ Additionally, it possesses anti-inflammatory and antioxidant properties.^468^ These multifaceted mechanisms collectively contribute to its efficacy in preventing AMS and HACE.	Although acetazolamide contains a sulfa moiety, the risk of an allergic reaction in those with sulfonamide allergies is extremely low.^469^ However, for individuals with a history of a severe allergic reactions to sulfonamide medications or Stevens-Johnson syndrome should avoid using acetazolamide. Moreover, it is contraindicated in cases of decreased sodium and/or potassium serum levels, severe kidney and liver diseases or dysfunctions, adrenal gland failure, hyperchloraemic acidosis, and cirrhosis.^369^	The primary side effect, arising from increased diuresis, is paraesthesias, particularly a “tingling” feeling in the extremities.^464^ Additionally, dizziness, hearing dysfunction or tinnitus, loss of appetite, taste alteration, gastrointestinal disturbances, as well as potential occurrences of kidney stones, myopia, drowsiness, and confusion may also be observed.^369^ Nevertheless, some of these adverse events can be minimized by using a low-dose regimen.	(1) Prevention: AMS 16 trials (randomized, placebo-controlled); 2301 participants (1255 with acetazolamide and 1046 with placebo).^369^ Acetazolamide effectively reduced AMS risk. HACE 6 trials (randomized, placebo-controlled); 1126 participants (586 with acetazolamide and 540 with placebo).^369^ Acetazolamide effectively reduced HACE risk. (2) Treatment: AMS 1 trial (randomized, placebo-controlled); 12 participants (6 with acetazolamide and 6 with placebo).^427^ Acetazolamide improved PaO_2_ and AMS. CMS 3 trials (randomized, placebo-controlled); 127 participants (82 with acetazolamide and 45 with placebo).^60,448,461^ Acetazolamide reduced erythrocytosis and improved pulmonary circulation without adverse effects.
Methazolamide	Methazolamide is a potent inhibitor of carbonic anhydrase. By enhancing systemic metabolic acidosis it improves ventilation and oxygenation levels, an effect comparable to that of acetazolamide, which is crucial in reducing the incidence and severity of AMS.^470^ The elevated oxygenation level consequently diminishes the production of ROS,^471^ alleviating cerebral edema and HPV,^470^ eliminating hypoxic fatigue, and mitigating excessive erythrocytosis.^449^ Beyond its role as a carbonic anhydrase inhibitor, methazolamide directly activates the antioxidant nuclear factor-related factor 2 (Nrf2) and inhibits the release of interleukin-1β (IL-1β).^472^ This carbonic anhydrase inhibition-independent antioxidative stress ability suggests that methazolamide may be more effective than acetazolamide in reducing ROS levels, positioning it as a superior option for the prevention and treatment of high-altitude illness.	Methazolamide is contraindicated in cases of decreased sodium and/or potassium serum levels, marked kidney and liver diseases or dysfunctions, adrenal gland failure, hyperchloraemic acidosis, and cirrhosis.^369^	There are several adverse reactions that may occur, especially early in the therapy.^369^ The most prominent one is paraesthesias, specifically a “tingling” feeling in the extremities. Additionally, hearing dysfunction or tinnitus; fatigue; malaise; loss of appetite; taste alteration; gastrointestinal disturbances such as nausea, vomiting, and diarrhoea; polyuria; and occasional instances of drowsiness and confusion may also be observed.	(1) Prevention: AMS 1 trial (randomized, placebo-controlled); 96 participants (29 with methazolamide and 67 with placebo).^373^ Methazolamide significantly lowered AMS incidence. (2) Treatment: AMS 1 trial (randomized, placebo-controlled); 15 participants (8 with methazolamide and 7 with placebo).^473^ Methazolamide notably improved AMS. CMS Animal study^449^
Dexamethasone	Dexamethasone, a synthetic glucocorticoid, is rapidly absorbed from the gastrointestinal tract. Its primary mechanisms of preventing high-altitude illness include reducing the permeability of cellular and capillary walls, decreasing alveolar inflammatory fluid leakage, inhibiting the formation of substances such as histamine, as well as suppressing hypoxia-induced cyclooxygenase-2 expression and related prostaglandin synthesis.^378^ Consequently, mitigates edema formation and ICP.^474^	It is contraindicated in patients with systemic fungal infections.^369^	Common side effects include hyperglycemia, fluid retention, hypokalemic alkalosis, potassium loss, and sodium retention.^378^ Additionally, dexamethasone has significant side effects on the hypothalamic-pituitary-adrenal axis. It may also elevate heart rate, posing a particular risk to those susceptible to HAPE.	(1) Prevention: AMS 8 trials (randomized, placebo-controlled); 226 participants (116 with dexamethasone and 110 with placebo).^378^ Dexamethasone could reduce the AMS incidence. (2) Treatment: AMS 1 trial (randomized, placebo-controlled); 35 participants (17 with dexamethasone and 18 with placebo group).^430^ Dexamethasone was more effective than placebo in treating AMS.
Ibuprofen	Non-steroidal anti-inflammatory drugs (ibuprofen, paracetamol, aspirin) alleviate or prevent high-altitude headaches and AMS by inhibiting cyclooxygenase to reduce prostaglandin production, thereby suppressing trigeminovascular sensitization and cerebral microvascular permeability.^475^	Ibuprofen is contraindicated in individuals with a known allergy to aspirin.^475^	It is prone to gastrointestinal bleeding with increased dosage and prolonged use of ibuprofen.^383^	(1) Prevention: AMS 3 trials (randomized, placebo-controlled); 437 participants (257 with ibuprofen and 180 with placebo).^379–381^ Ibuprofen effectively prevented AMS.
Nifedipine	Since excessive HPV induces HAPE, nifedipine, a Ca2+ channel blocker, can reduce pulmonary vascular resistance by inbihiting Ca2+ influx, thus relieving pulmonary hypertension.^219^	Nifedipine should not be administered to patients with cardiogenic shock as well as with known hypersensitivity to any of its components.^369^	Common side effects associated with nifedipine include headache, flushing/heat sensation, dizziness, fatigue/asthenia, and nausea.^369^ Additionally, although hypotension is generally not a concern with the extended-release formulation, it may occur in a few individuals.^20^	(1) Prevention: HAPE 1 trial (randomized, placebo-controlled); 21 participants (10 with nifedipine and 11 with placebo).^384^ Nifedipine lowered PAP and prevented HAPE. (2) Treatment: HAPE 2 trials (randomized, placebo-controlled); 201 participants (96 with supplemental O_2_ and nifedipine 115 with supplemental O_2_ and placebo).^437,439^ Oxygen alone was adequate therapy for HAPE and that adjuvant pharmacotherapy with nifedipine did not hasten recovery.
PDE5 inhibitors (tadalafil and sildenafil)	Selective inhibitors of PDE5, such as tadalafil and sildenafil, can induce overproduction of NO, which, in turn, attenuates HPV, leading to a reduction in pulmonary hypertension.^476^	If a subject is using any form of organic nitrate regularly or intermittently, the PDE5 inhibitor is contraindicated as it potentiates the hypotensive effects of nitrates.^369^	The common side effects include headaches and flushing.^369^	(1) Prevention: HAPE 1 trial (randomized, placebo-controlled); 17 participants (8 with tadalafil and 9 with placebo).^231^ Tadalafil effectively prevented HAPE in susceptible individuals. HAPH Animal study^402–404^ (2) Treatment: HAPE 1 trial (randomized); 10 participants (7 with sildenafil and nifedipine while 3 with nifedipine alone).^435^ Limited case series suggested the therapeutic effect of sildenafil on HAPE. HAPH 1 trial (randomized, placebo-controlled); 17 participants (9 with sildenafil and 8 with placebo).^455^ Sildenafil lowered PAP and improved HAPH.
Salmeterol	Salmeterol has high affinity to the human β2 adrenergic receptor (B2AR), which contributes to the reduction in HPV and PAP, thus relieving pulmonary hypertension.^477^ Additionally, salmeterol enhances alveolar clearance by stimulating amiloride-sensitive sodium (Na) channels, a mechanism that likely improves HAPE occurrence and progression.^478^	It is contraindicated in asthmatic patients and should be used with caution in those with cardiovascular disorders.^369^	The side - effects include an increased risk of asthma-related death and a range of symptoms such as seizures and abnormal blood pressure.^369^	(1) Prevention: HAPE 1 trial (randomized, placebo-controlled); 37 participants (18 with salmeterol and 19 with placebo).^258^ Salmeterol reduced HAPE incidence but often caused tremor and tachycardia at high dose.

##### AMS and HACE

*Carbonic anhydrase inhibitors: acetazolamide and methazolamide*

*Acetazolamide:* As the primary prophylactic agent for AMS and HACE, the efficacy of acetazolamide has been well-established in numerous trials.^367^ The WMS 2024 guidelines designate acetazolamide as the first-line prophylaxis for moderate-to-high-risk individuals.^20^ The appropriate dosage of acetazolamide for preventing AMS and HACE has been a subject of debate.^367,368^ Systematic reviews confirmed that it effectively reduced AMS risk (RR = 0.47; 16 trials; 2301 participants; moderate quality of evidence) and HACE risk (RR = 0.32; 6 parallel trials; 1126 participants; moderate quality of evidence), but increased paraesthesia risk.^369^ The WMS 2024 guidelines strongly recommend starting 125 mg twice daily for adults (2.5 mg/kg for children) one day before ascent, and continuing until descent.^20^ This dosage may be inadequate for extremely rapid ascent or altitudes >5000 m. High-risk climbers may consider 250 mg every 12 h, although dosing >5000 m remains uncertain.

*Methazolamide:* This lipophilic acetazolamide analog exhibits remarkable advantages over acetazolamide in preventing AMS due to its higher lipid solubility, lower plasma protein binding, reduced renal excretion, and fewer side effects.^370^ Two clinical trials suggested that lower-dose methazolamide (100–200 mg daily) had similar prophylactic efficacy comparable to higher dose of acetazolamide (500 mg daily).^371,372^ However, the limited number of subjects in these trials (~10 subjects in each group) undermined the conclusions. A recent larger-sample trial demonstrated the significant efficacy of methazolamide in reducing the AMS incidence: compared with placebo (n = 38), methazolamide (n = 29; 50 mg per dose, twice a day for 6 days, starting 2 days before ascent) significantly lowered AMS incidence.^373^ According to the above trials, we recommend a dosage of methazolamide for AMS prevention of 50 mg twice daily. Considering its advantages over acetazolamide, methazolamide has emerged as a potential alternative for AMS and HACE prophylaxis. Nonetheless, larger samples and higher-quality trials are required to thoroughly evaluate its efficacy.

*Corticosteroids: dexamethasone*

Dexamethasone, a well-researched alternative for those intolerant of or contraindicated with acetazolamide, effectively prevents AMS/HACE despite not facilitating acclimatization as effectively as acetazolamide.^374–377^ Generally, the recommended dosage is 2 mg every 6 h or 4 mg every 12 h.^11^ For high-risk scenarios (e.g., rapid military ascent >3500 m with exertion), 4 mg every 6 h may be considered, although this should be an exception.^20^ Owing to the risk of adrenal suppression, dexamethasone use should be limited to ≤7 days.^351^ If longer-term use is deemed necessary, gradual tapering of the dosage is recommended. Notably, two meta-analyses have yielded conflicting results regarding dexamethasone’s ability to prevent AMS. On the basis of eight studies comparing dexamethasone with placebo, Tang et al. reported a significant reduction in AMS incidence, with an odds ratio of 6.03.^378^ In contrast, Estrada’s meta-analysis, encompassing six parallel trials and five crossover studies, failed to provide definitive evidence of dexamethasone’s effectiveness, rating the quality of this evidence as low.^369^ Thus, dexamethasone should be prescribed only by experienced high-altitude physicians.

*Non-steroidal anti-inflammatory drugs: ibuprofen*

Ibuprofen is generally a second-line AMS prophylactic. It effectively prevents high-altitude headache and AMS, but does not facilitate acclimatization and is less effective than acetazolamide. Multiple trials indicated that it outperforms placebo,^379,380^ whereas comparisons with acetazolamide have yielded contradictory results. One trial indicated similar efficacy in preventing high-altitude headaches and AMS in the acetazolamide and ibuprofen groups.^381^ Another trial revealed that ibuprofen was inferior to acetazolamide.^382^ For those declining or intolerant of acetazolamide/dexamethasone, ibuprofen is an alternative. The recommended daily dosage of ibuprofen ranges from 600 to 1800 mg.^369^ However, this recommendation is based on moderate-quality evidence, resulting in a weak recommendation. Moreover, the efficacy and safety of prolonged ibuprofen use at high altitudes remain uncertain, with potential risks such as gastrointestinal bleeding or renal dysfunction.^383^

##### HAPE

*Ca*^2+^ *antagonist: nifedipine*

Given that excessive HPV contributes to HAPE pathogenesis, the pulmonary vasodilator nifedipine stands out as the primary medication for HAPE prevention. As a Ca^2+^ channel blocker, nifedipine reduces Ca^2+^ influx to alleviate HPV, thereby decreasing pulmonary vascular resistance and PAP without causing significant hypotension.^219^ A single, randomized, placebo-controlled study confirmed that nifedipine lowered PAP and prevents HAPE.^384^ The WMS 2024 guidelines recommend 30 mg every 12 h, which was initiated 24 h prior to ascending and continued until either descent begins.^20^ It is preferred for individuals with a history of HAPE, especially recurrent episodes. PDE5 inhibitors (tadalafil or sildenafil) and the β2-receptor agonist salmeterol are also potential options.

*PDE5 inhibitors and β2 agonists*

*PDE5 inhibitors:* PDE5 inhibitors (tadalafil or sildenafil) play unique roles in preventing HAPE. For nifedipine-intolerant travelers, tadalafil administration has emerged as an alternative preventive measure. A single, randomized, placebo-controlled trial indicated that tadalafil 10 mg every 12 h effectively prevented HAPE in susceptible individuals, despite the small sample size and two withdrawals due to severe AMS.^231^ Although only tadalafil has been investigated, it is reasonable to assume that sildenafil could be similarly effective. Since tadalafil lacks sufficient clinical experience, more data are needed before tadalafil can be recommended as a nifedipine alternative. Importantly, due to the possible occurrence of severe hypotension, combined therapy or prophylaxis with nifedipine and PDE5 inhibitors should be avoided.

*Salmeterol:* One randomized, placebo-controlled study demonstrated that inhaled salmeterol (125 μg twice daily) reduced HAPE incidence by 50% in susceptible individuals, but this high dosage was often associated with tremor and tachycardia.^258^ Owing to its lower effectiveness compared to Ca^2+^ antagonist and PDE5 inhibitors, along with limited clinical experience and insufficient data, salmeterol is not recommended for HAPE prevention.^351^

##### Traditional Chinese medicines (TCMs) in preventing acute altitude illness

Two trials suggested *Ginkgo biloba* may prevent AMS,^385,386^ but others reported negative results,^387,388^ potentially due to product variability.^389^ Furthermore, *Ginkgo biloba* is associated with serious bleeding and adverse cardiac events, and its long-term safety in humans remains unclear due to rodent toxicity/cancer concerns.^390^ Therefore, it is not recommended for AMS prophylaxis. *Rhodiola crenulata* failed to reduce AMS risk in a crossover study (n = 125; 800 mg/day at 3421 m vs. placebo).^391^ In the Andes, chewing *coca* leaves, drinking *coca* tea and using other *coca*-derived products are common methods for preventing AMS,^392^ but no substantial evidence supports this practice. In addition to clinical trials, some TCMs, such as *Rhodiola rosea*,^393^ *Hippophae rhamnoides*,^394^ *Aesculus chinensis*,^395^ *Portulaca oleracea Linn* (*Portulacaceae*),^396^ *Phlomis younghusbandii Mukerj* (*Lamiaceae*),^397^ *Pueraria lobata*,^398^ *Eleutheroside B*,^399^ and *Notoginsenoside R1*,^400,401^ showed promise in preventing HAPE/HACE in rat models by inhibiting the infiltration of inflammatory cells, the production of inflammatory cytokines, and apoptosis/pyroptosis. In particular, *Pueraria lobata* prevented hypobaric hypoxia-induced lung/brain injury in rats by downregulating inflammatory cytokines, AQP1, AQP4, and NF-κB signaling pathway.^398^ Medicinal plants are increasingly considered as supplementary or alternative medications for preventing acute high-altitude diseases, but most evidence is empirical or preclinical. Given the insufficient, low-quality, and/or conflicting evidence in current knowledge, their routine use for the prevention of acute altitude illness is not recommended.

##### Chronic altitude illness

Although medications have been successful in preventing acute altitude illness, specific early warning, predictive, and preventive measures for chronic high-altitude hypoxia-related diseases are lacking worldwide. Clinically, no specific preventive drugs exist. Only sildenafil (a PDE5 inhibitor) and some TCMs show potential preventive effects in HAPH on animal models, which requires clinical validation. Recent studies demonstrated that sildenafil exhibited significant efficacy in preventing HAPH.^402–404^ By modulating PPARγ/TRPC1/TRPC6 expression or inhibiting Notch3 signaling, sildenafil effectively mitigated PASMCs proliferation and HPVR, alleviating right ventricular pressure/hypertrophy.^403,404^

Additionally, TCMs play pivotal roles in HAPH prevention: *Danshensu*,^405^ *Oxymatrine*,^406^ *Salvia Przewalskii Maxim*,^407^ and *Hydroxysafflor Yellow A*^408^ mitigated HPVR and right ventricular hypertrophy by suppressing inflammatory responses and PASMCs proliferation. Traditional Tibetan medicines have demonstrated unique advantages in HAPH prevention. *Luteolin* alleviated HPVR via the HIF-2α-Arg-NO axis and the PI3K-AKT-eNOS-NO signaling pathway, protecting PAECs function, with efficacy comparable to that of sildenafil.^409^ It also upregulated Kv1.5 to reduce PASMC proliferation/promote apoptosis, thus alleviating HPVR.^410^ *Tsantan Sumtang* mitigated hypoxia-induced pulmonary remodeling to prevent HAPH by protecting endothelial function and inhibiting PASMCs proliferation.^411–414^ *Rhodiola* and its active fractions (such as bioactive fraction from *Rhodiola algida* (ACRT) and volatile oil of *Rhodiola tangutica* (VORA)) also exhibited preventive effects on HAPH through inhibiting PASMCs proliferation by equilibrating the ACE-AngII-AT1R/ACE2-Ang1-7-Mas axis; reducing proliferating cell nuclear antigen (PCNA), cyclin D1, CDK4 expression; or suppressing p27Kip1 degradation.^415–417^ *Echinacoside*,^418^ *Srolo Bzhtang*,^419^ *Kaempferol*,^420^ and *4-Terpineol*^421^ also improved pulmonary artery remodeling in HAPH rats by modulating the MAPK/NF-κB signaling pathway in PAECs/PASMCs.

### Treatment of altitude illness

#### AMS and HACE

AMS and HACE are serious conditions requiring urgent management.^9,20,351,352^ When AMS is suspected or diagnosed, the immediate cessation of ascent is crucial to prevent further deterioration. For mild–moderate AMS, individuals should stay at the current altitude with symptom monitoring. Symptomatic treatment (nonopioid analgesics for headaches, antiemetics for nausea/vomiting) is often sufficient.^422^ However, if symptoms persist/worsen after 1–3 days, descent to a lower altitude becomes necessary.^20^ HACE demands immediate descent. If descent is not feasible, supplemental oxygen or a portable hyperbaric chamber should be used. Dexamethasone is recommended for both moderate-severe AMS and HACE, whereas acetazolamide may be added for AMS.^11,20^ Traditional Tibetan medicines (*Rhodiola* and *Tibetan turnip*) show promising effects in treatment of acute altitude illness.

##### Descent

Descent remains the single best treatment measure for AMS and HACE and has proven effective across all severities, particularly for severe cases, with strong evidence supporting this recommendation.^20,351^ However, its applicability is not absolute and must be assessed in the context of terrain, weather conditions, and the presence of injuries. Generally, severe AMS or HACE patients are advised to descend until their symptoms fully resolve, a process that typically involves a drop in altitude ranging from 300 to 1000 m.^20,351^

##### Supplemental oxygen

Although clinical trials are limited,^423^ extensive clinical experience suggests that supplemental oxygen is an effective measure to alleviate symptoms when descent is impractical or pending.^20^ Typically, oxygen is administered via a nasal cannula or mask at flow rates sufficient to alleviate symptoms, aiming for an SpO_2_ above 90%, which is generally deemed adequate. The use of low-flow oxygen (1–2 L/min) for at least 2 h has greater benefits than short bursts of large amounts of oxygen.^20^ Similarly, for patients with HACE, low-flow oxygen (2–4 L/min) is recommended to alleviate symptoms, albeit with limited research.^352^

##### Portable hyperbaric chambers

In severe AMS and HACE, if descent and oxygen are unavailable, portable hyperbaric chambers emerge as an effective treatment option.^20^ Hyperbaric therapy effectively mimics a descent in altitude, thereby increasing arterial oxygenation to alleviate symptoms of acute altitude illness. A limited number of clinical trials and case reports demonstrated that 1 h of compression with 193 mbar in the hyperbaric chamber, corresponding to a descent of 2250 m, lead to a short term improvement in symptoms of AMS but had no long–term beneficial effect.^424–426^ Despite several limitations and the risk of recurrence, their use in emergency situations should not deter.

##### Carbonic anhydrase inhibitors: acetazolamide and methazolamide

*Acetazolamide:* While proven effective for preventing AMS, evidence supporting its use for treatment is limited to a single small-scale study.^427^ This study indicated that administering 250 mg acetazolamide upon arrival and again at 8 h was more effective than the placebo. Thus, for patients who are unresponsive to symptomatic treatments, some experts suggest considering a dosage of 250 mg of acetazolamide twice daily.^20,351^ However, whether a lower dose is sufficient remains unknown. Robust evidence regarding the optimal dosage for AMS treatment and its effectiveness in HACE patients is currently lacking.^427^ Therefore, acetazolamide is not routinely recommended for treating AMS and HACE.

*Methazolamide:* A randomized double-blind trial suggested that methazolamide had comparable efficacy to acetazolamide for AMS treatment.^428^ AMS patients administered placebo experienced notable worsening of symptoms, whereas those treated with acetazolamide (1.5 g daily) improved. Strikingly, treatment with a lower dose of methazolamide (daily dose of 200 mg) also resulted in notable improvements in AMS symptoms, particularly headache, irritability, feeling unwell and loss of concentration. However, definitive superiority could not be established due to insufficient control data from one expedition. More detailed clinical studies are warranted to thoroughly evaluate the early response of AMS patients to methazolamide.

##### Corticosteroids: dexamethasone

Dexamethasone has demonstrated remarkable efficacy in treating AMS and HACE. Controlled studies showed that an initial 8 mg dose, followed by 4 mg every 6 h until all symptoms resolve, was more effective than placebo in treating AMS.^374,429,430^ While specific research on combining acetazolamide and dexamethasone was lacking, experts suggested considering this combination, especially for moderate-to-severe AMS.^351^ Regarding HACE patients, although no dedicated studies have been conducted, clinical experience has supported a similar regimen (8 mg initially, then 4 mg every 6 h).^20^

##### Traditional Tibetan medicines

Traditional Tibetan medicines, particularly *Rhodiola rosea*, show unique efficacy against AMS. A randomized, single-blind, placebo-controlled trial revealed that compared with placebo, *Rhodiola rosea* capsules significantly alleviated symptoms such as fatigue, drowsiness, chest tightness, palpitations, vertigo, inattention, and memory loss among 543 soldiers, participating in earthquake relief on the plateau.^431^ Mechanistic studies suggested that combining *Rhodiola* with acetazolamide could downregulate HIF-1α expression and improve hemodynamics in rats, thereby reducing the body’s sensitivity to acute hypoxia.^432^ Overall, *Rhodiola rosea* is promising for AMS treatment and has synergistic potential with acetazolamide, warranting more comprehensive clinical investigations.

#### HAPE

HAPE is a severe acute high-altitude disease that requires immediate action. Suspected or diagnosed HAPE warrants immediate oxygen administration (if available) and prompt descent to lower altitudes.^11,20,433^ If descent is impossible or delayed, supplemental oxygen or a portable hyperbaric chamber should be used. In well-resourced medical settings, oxygen therapy with monitoring at the current altitude may suffice, but descent is necessary if oxygen and continuous positive airway pressure (CPAP) fail or if the patient deteriorates. In resource-limited field settings, nifedipine and PDE5 inhibitors can serve as adjuncts, but the concurrent use of multiple pulmonary vasodilators is discouraged.^434,435^ β2 agonists and diuretics lack supporting data and are not recommended.^435^ Overall, descent is the best treatment option for HAPE, but in specific circumstances, oxygen therapy and pulmonary vasodilators may alleviate symptoms.

##### Descent

Descending to a lower altitude is the most effective treatment for HAPE.^20,351^ Patients should descend at least 1000 m as soon as possible until symptoms improve. Excessive exertion during descent (using vehicles, helicopters, animals, or walk without heavy loads) should be avoided, as it can exacerbate this condition. The duration of symptom relief varies, depending on patients’ physical condition, illness severity, and adaptability. Symptoms typically gradually subside after reaching the target altitude.

##### Supplemental oxygen

If descent is impossible or delayed, supplemental oxygen should be started immediately via a nasal cannula or mask to achieve SpO_2_ > 90%. Two clinical studies demonstrated that HAPE patients could be safely managed with bed rest and supplemental oxygen without descent.^436,437^ Recent evidence recommended high-flow oxygen (8–10 L/min for 24 h), followed by 4–6 L/min until symptoms subside, and then 2 L/min for at least 12 h daily until symptoms disappeared.^437^ Importantly, compared with oxygen and bed rest alone, adjuvant therapy with dexamethasone or nifedipine did not accelerate recovery.

##### Portable hyperbaric chambers

Portable hyperbaric chambers are useful when descent or when oxygen is unavailable. Evidence is primarily anecdotal (case reports). In a specific case, after being placed in a portable hyperbaric chamber and having the oxygen delivery rate reduced from 3 L/min to 0.5 L/min for ~8 h, the patient experienced some relief of symptoms but ultimately required helicopter evacuation to the nearest hospital for further care.^438^

##### Calcium antagonists: nifedipine

Nifedipine is a backup option under extremely restricted conditions.^437,439^ A single non-randomized, unblinded study indicated that a regimen of 10 mg short-acting initially, followed by 20 mg slow-release every 6 h, could be effective when oxygen or descent was unavailable.^437^ However, the combination of nifedipine and supplemental oxygen did not provide significant advantages over supplemental oxygen alone. Based on these findings and clinical experience, the WMS 2024 guidelines recommend nifedipine (30 mg sustained release twice daily) only when descent is impossible/delayed and oxygen is unavailable.^20^

##### Alternative options based on case series

*CPAP:* CPAP is theorized to benefit acute altitude illness by the increasing arterial partial pressure of oxygen through augmenting transmural pressure across alveolar walls, thus expanding alveolar volume, optimizing ventilation–perfusion matching, and improving gas exchange. Only two case reports described the use of CPAP/EPAP in HAPE treatment.^440,441^ Due to the absence of randomized controlled trials, the precise therapeutic effectiveness of CPAP in HAPE remains unsystematically validated. Overall, it is weakly recommended to consider using CPAP for HAPE treatment if oxygen or pulmonary vasodilators are unavailable or ineffective.^20^

*PDE5 inhibitors (tadalafil/sildenafil):* PDE5 inhibitors possess a strong physiologic rationale for HAPE treatment due to their ability in pulmonary vasodilation. Limited case series have suggested the therapeutic effect of sildenafil on HAPE.^435^ Given the limited evidence, some experts suggest considering tadalafil (10 mg) or sildenafil (50 mg) only if descent is impossible and oxygen as well as nifedipine are unavailable.^351^ Additionally, the combined use of nifedipine and PDE5 inhibitors should be avoided due to the risk of hypotension.

*β2 agonists and diuretics:* Although salmeterol has been reported to be used in the treatment of HAPE with potentially low risk, there are currently no definitive data supporting its benefits in HAPE patients.^435^ Meanwhile, systematic studies evaluating the role of β2 agonists, such as salmeterol, in HAPE treatment are lacking. Diuretics play no role in HAPE treatment, particularly considering the intravascular volume depletion often present in many HAPE patients.^442^ Therefore, routine use is strongly discouraged.

#### CMS

CMS presents a significant threat to the health and life quality of high-altitude residents. Currently, its treatment methods can be divided into two major categories: non-pharmacological management and pharmacological treatment. The traditional and definitive treatment for CMS is descent to lower altitudes or sea level; however, this approach is only a temporary solution unless the patient permanently relocates to a lower altitude.^443^ Bloodletting therapy, another non-pharmacological management in traditional Tibetan medicine, is a commonly used palliative measure. It reduces RBC mass and partially ameliorates the signs and symptoms of CMS.^444^ However, Hct may also rebound after bloodletting, and frequent use can lead to metabolic disorders, making it an impractical long-term treatment. Pharmacological management involves the use of carbonic anhydrase inhibitors such as acetazolamide, methazolamide, and natural products, such as *Lepidium meyenii* (*maca*) and *Duoxuekang*.

##### Non-pharmacological management

*Descent*

Descending to lower altitudes has emerged as the most definitive approach for CMS treatment. Upon relocation to lower altitudes or sea level, prompt alleviation of subjective symptoms and sleep disorders can be observed, accompanied by the disappearance of alveolar hypoxia, hypoxemia, and cyanosis.^443^ Polycythemia diminishes, with Hb and Hct levels reverting to sea level norms within a few weeks to months. Pulmonary hypertension and right ventricular hypertrophy gradually reverse and disappear within one to two years. However, the clinical manifestations of CMS reappear when the patient returns to a high altitude.^445^ It is merely a temporary solution unless the patient permanently relocates to a lower altitude. In severe cases, despite the impracticality due to social, familial, and economic constraints, permanent relocation to a lower altitude is advisable.

*Bloodletting*

Bloodletting, encompassing both standalone bloodletting and isovolemic hemodilution, serves as a palliative measure to reduce polycythemia and improve some symptoms of CMS.^286^ Distinct Tibetan bloodletting techniques involve small-volume blood removal (50–100 mL), combined with herbal preparations such as “Three Fruit Soup”, which are aimed at separating “bad blood”.^23^ Routine phlebotomies, with or without concurrent volume replacement, are often used to normalize the RBC mass and Hb concentration relative to the altitude of residence.^269,444,446^ However, this approach involves three primary concerns: (1) Frequent procedures may cause metabolic disorders, exertional dyspnea, and fatigue, and clinical trials to assess its safety and efficacy are lacking; (2) It can induce iron deficiency, subsequently elevating PAP and exacerbating pulmonary hypertension^447^; and (3) Increases in Hct and symptom recurrence are common. Owing to its transient effects, invasiveness, and potential adverse impacts, bloodletting is not recommended as a long-term treatment for CMS.

##### Pharmacological management

*Carbonic anhydrase inhibitors: acetazolamide and methazolamide*

*Acetazolamide:* Currently, acetazolamide treatment is considered one of the most effective pharmacological strategies for CMS treatment. Acetazolamide can significantly reduce Hct and serum EPO levels in CMS patients, while simultaneously improving nocturnal oxygen saturation, decreasing the frequency of apnea-hypopnea episodes, and lowering pulmonary vascular resistance.^60,448^ The typical oral dose is 250 mg daily, which is used for periods ranging from 3 weeks to 6 months. While effective within weeks, reductions in pulmonary vascular resistance are more pronounced after longer treatment (e.g., 6 months).^448^ In summary, acetazolamide appears to be an efficient and safe option for chronic CMS treatment, although its long-term effects need to be further addressed.

*Methazolamide:* Although clinical trials of methazolamide in treating CMS are limited, animal studies revealed that it reduced Hb concentration, Hct, and blood viscosity in a dose-dependent manner.^449^ Notably, a dosage of 10 mg/kg/day of methazolamide significantly improved these parameters, exhibiting similar efficacy to 30 mg/kg/day of acetazolamide. Mechanistically, methazolamide enhanced the Hct-to-viscosity ratio to improve the oxygen delivery capacity. Conclusive evidence of its efficacy and safety in humans requires additional clinical trials.

*Natural medicines*

Natural medicines also exhibit promising efficacy in CMS treatment. *Lepidium meyenii*, known as *maca*, has been utilized as a dietary supplement to enhance the health of highlanders. Daily consumption of 3 g of spray-dried *maca* extracts (either red or black) for 12 weeks significantly reduced the CMS score and Hb concentration.^450^ Red *maca* may be superior to black *maca* for lowering Hb level and improving CMS symptoms. However, further studies are needed to confirm the effects and mechanisms of different types of *maca* for treating CMS. *Duoxuekang* capsule, a traditional Tibetan medicine composed of four herbs (*Phyllanthus emblica*, *Rhodiola crenulata*, *Hippophae rhamnoides*, and *Zingiber officinale*), was administered orally at a dosage of four capsules daily for four weeks among 14 CMS patients.^451^ After treatment, the patients’ symptoms significantly improved, and the levels of RBCs, Hb, and Hct decreased while the oxygen saturation increased.

#### HAPH

HAPH poses a severe health risk to high-altitude residents, making effective treatment crucial. The ideal management is permanent relocation to lower altitudes.^452–454^ For patients who choose to remain at high altitudes, several drugs have been studied for HAPH treatment, yet their efficacy and applicability require further assessment. Although acetazolamide has not been specifically studied for HAPH, it can reduce hypoventilation, improve pulmonary circulation, and decrease pulmonary vascular resistance in CMS treatment. Sildenafil has shown promising benefits for HAPH patients by blocking the breakdown of NO to promote pulmonary artery vasodilation.^455^ Conversely, the effect of bosentan remains controversial, warranting more comprehensive investigations in large population cohorts.^456,457^ Additionally, *Rhodiola*^458,459^ and *fasudil*^460^ also show potential in treating HAPH.

##### Non-pharmacological management

Currently, there are limited data available to support the long-term management of HAPH. The primary recommendation for HAPH patients is to relocate to lower altitudes. Two clinical studies indicated that the mPAP of HAPH patients could normalize after residing at lower altitudes.^452,454^ However, mPAP increased again if patients returned to high altitude.^453^

##### Pharmacological management

*Carbonic anhydrase inhibitor: acetazolamide*

While not specifically studied for HAPH, acetazolamide has beneficial effects on CMS, including reducing pulmonary vascular resistance and improving nocturnal oxygen saturation.^60,448,461^ These results suggest that acetazolamide may also play an important role in the treatment of HAPH. Given its favorable performance in related conditions, favorable side-effect profile, and low cost, it is expected to become an important option for HAPH treatment.

*PDE5 inhibitor: sildenafil*

Sildenafil has demonstrated therapeutic efficacy in HAPH patients. Short-term use of sildenafil (1–2 days) could significantly reduce sPAP, although its impact on oxygen saturation and heart rate at high altitudes was not significant.^462^ Compared with placebo, long-term administration (e.g., 25 mg or 100 mg every 8 h for 12 weeks) significantly decreased mPAP and improved the 6 min walk distance, with good tolerability.^455^ Therefore, sildenafil holds promise as a therapeutic option for HAPH patients, warranting further investigation to address its efficacy and safety.

*ET-1 antagonism: bosentan*

Bosentan, an ET-1 receptor antagonist, has been applied in the treatment of HAPH. While it is effective in treating pulmonary arterial hypertension at sea level, the results at high altitudes are inconsistent.^463^ Seheult et al. reported that it was ineffective in preventing exercise-induced desaturation or reducing sPAP during acute hypoxia exposure in healthy individuals, even worsening oxygen desaturation during intense exercise.^456^ Conversely, Kojonazarov et al. reported that a single bosentan dose (125 mg for 3 h) reduced sPAP more effectively than oxygen therapy in 15 HAPH patients.^457^ This discrepancy may reflect differences in the study populations: Kojonazarov et al. studied the therapeutic effects of bosentan in HAPH patients, whereas Seheult et al. evaluated its preventive role in healthy individuals exposed to acute hypoxia. Therefore, bosentan may have therapeutic potential in established HAPH, but its role in HAPH prevention remains uncertain. Nevertheless, more comprehensive clinical trials are essential to determine the long-term efficacy and safety of bosentan in HAPH.

*Chinese medicinal plants*

In recent years, certain Chinese medicinal plants have shown potential therapeutic effects on HAPH. *Rhodiola*, a traditional Tibetan medicine, has attracted considerable attention for its therapeutic effects on HAPH. Compared with conventional therapy alone, the oral administration of *Rhodiola* (2.0 grams three times daily for three weeks) significantly reduced serum basic fibroblast growth factor (bFGF) levels and mPAP in HAPH patients.^458^ Mechanistically, *Rhodiola* may improve pulmonary hypertension by inhibiting ET-1 and increasing NO synthesis/release.^459^ Despite promising results, further research is needed to confirm the efficacy, safety, and optimal dosage in HAPH treatment.

*Others*

Kojonazarov et al. conducted a double-blind randomized study on the Rho A/Rho kinase inhibitor fasudil (1 mg/min for 30 min, with a total dose of 30 mg), involving 19 HAPH patients who are permanent residents of the Tien-Shan Mountains at an altitude of 3200–3600 m.^460^ Intravenous fasudil markedly reduced sPAP without affecting systemic blood pressure in HAPH patients, demonstrating good tolerability (minor side effects: facial flushing, dry mouth). Smith et al. explored the relationship between iron availability and HAPH.^447^ Interestingly, iron infusion was associated with reduced sPAP in sea-level residents exposed to acute hypoxia at 4340 m, whereas iron infusion cannot improve sPAP among CMS patients with phlebotomy. Thus, iron supplementation may be ineffective in HAPH treatment. Definitive conclusions regarding iron manipulation (supplementation or depletion) in HAPH require more clinical trials.

## Conclusions and perspectives

High-altitude hypoxia induces complex physiological changes that drive adaptations or maladaptations, culminating in hypoxemia and various high-altitude illnesses. This review delineates the pathogenesis and interventions for altitude illnesses, highlighting the complexity of high-altitude illnesses. In terms of epidemiology, the reported prevalence of altitude illnesses varies significantly due to differences in sample size, ethnic population, diagnostic methods/criteria, study design and environmental conditions. More comprehensive and standardized studies are needed to accurately determine the true prevalence and risk factors associated with these diseases.

Genetic susceptibility plays a crucial role in altitude illnesses, yet our understanding remains limited. The diagnosis of AMS involves a complex scoring of clinical symptoms, incorporating subjectivity and intricate pathophysiology, which consequently leads to heterogeneity in genetic association studies. Revising the diagnostic criteria for AMS to include objective clinical indicators is essential. Additionally, when collecting AMS cases, stratifying them based on symptom presentations or underlying pathology enables group-specific research into genetic mechanisms associated with these symptoms. The genetic architecture of HAPE and HAPH remains largely unknown due to diverse sample sizes and population backgrounds. A cost-effective approach can be devised by first selecting tests on the whole-genome sequencing (WGS) of a smaller cohort, followed by genotyping and association tests on a larger cohort. Genes identified through this process would then be considered for further validation.

Despite significant advancements, there are many unanswered questions about the pathophysiological mechanisms of symptoms in high-altitude illness, and several existing mechanisms are still a matter of debate. In AMS and HACE, the roles of cerebral hemodynamics and fluid alteration require further exploration. HAPE pathogenesis involves multiple factors, but the precise contributions and mechanisms of inflammatory responses are not fully understood. For CMS, the currently identified potential pathogenic mechanisms only show a negative correlation with CMS, lacking direct evidence of causality. Future studies should expand the sample size, comprehensively collect clinical indicators, identify pathogenic variants, and apply the Mendelian randomization (MR) method to predict causal relationships, with experimental verification as the final step. In HAPH, although key processes such as hypoxic pulmonary vasoconstriction and vascular remodeling have been identified, the underlying mechanisms and the roles of various mediators need further clarification.

Strategies such as gradual ascent, preacclimatization, descent, and several pharmacological agents (e.g., acetazolamide, methazolamide, dexamethasone, nifedipine, and sildenafil) are widely accepted as effective strategies for preventing and treating acute altitude illnesses. However, the options for treating chronic altitude sicknesses, such as CMS and HAPH, are limited, which pharmacological interventions are largely experimental. Further research into novel preventive and therapeutic strategies is urgently needed, especially for CMS and HAPH. Additionally, exploring the potential of natural medicines, such as traditional Tibetan medicines, could identify novel and effective management strategies for chronic altitude illness.

## Data Availability

Not applicable.
